# Genetic diversity, admixture, and hatchery influence in Brook Trout (*Salvelinus fontinalis*) throughout western New York State

**DOI:** 10.1002/ece3.5237

**Published:** 2019-06-14

**Authors:** Stephanie Dowell Beer, Scott Cornett, Peter Austerman, Betsy Trometer, Thomas Hoffman, Meredith L. Bartron

**Affiliations:** ^1^ U.S. Fish and Wildlife Service Northeast Fishery Center Lamar Pennsylvania; ^2^ New York State Department of Environmental Conservation Allegany New York; ^3^ New York State Department of Environmental Conservation Avon New York; ^4^ U.S. Fish and Wildlife Service Lower Great Lakes Fish and Wildlife Conservation Office Basom New York

**Keywords:** effective population size, gene flow, genetic structure, hatchery introgression, relatedness, salmonid

## Abstract

Although Brook Trout are distributed across most of eastern North America, population numbers have declined in many regions due to habitat loss, climate change, and competition with non‐native species. In New York State, Brook Trout habitat has been substantially reduced, with many areas showing complete extirpation of Brook Trout populations, predominantly in the western portion of the state. Small, fragmented populations are at risk of genetic diversity loss, inbreeding depression, and reduced fitness, leading to a greater potential for local extirpation. Genetic monitoring is a practical tool that can facilitate further conservation‐decision making regarding small populations. In this study, we used 12 microsatellite loci to examine 3,436 sampled Brook Trout, representing 75 sites from the Allegheny, Erie/Niagara, Genesee, Oswego, Lake Ontario, and Susquehanna drainage basins throughout western New York State. Three Brook Trout hatchery strains were also genetically characterized to evaluate the degree of hatchery introgression between wild populations and hatchery strains stocked in the region. Overall, estimates of genetic diversity varied widely: Allelic richness ranged from 2.23 to 7.485, and expected heterozygosity ranged from 0.402 to 0.766. As observed for Brook Trout in other regions, we found a high degree of genetic differentiation among populations, with all comparisons except one showing significant *F*_ST _values. Hatchery introgression was found to be minimal, with estimates ranging from 1.96% to 3.10% of wild individuals exhibiting membership proportions to a hatchery strain cluster exceeding 10% (*q* ≥ 0.10). Results from this investigation can be used to prioritize management efforts for Brook Trout in western New York State and act as a baseline to monitor future population trends.

## 1 INTRODUCTION

As the only stream‐dwelling salmonid native to the eastern United States, Brook Trout (*Salvelinus fontinalis*) are an iconic species, representative of pristine headwater streams, and valued for their recreational and economic importance (Eastern Brook Trout Joint Venture, 2011). The distribution of Brook Trout spans from northern Quebec though Georgia and extends west to include all of the Great Lakes and part of the upper Mississippi (MacCrimmon & Campbell, 1969; Scott & Crossman, 1973). Although this species was historically common in most cold‐water streams and rivers throughout this range (MacCrimmon & Campbell, 1969), during the past century, Brook Trout populations have substantially declined in many regions. In the eastern United States portion of their range, Brook Trout are considered extirpated from 41% of subwatersheds and are greatly reduced in another 51% of subwatersheds (Eastern Brook Trout Joint Venture, 2016). Brook Trout populations have been negatively affected by a variety of factors, including land conversion and agriculture (Hudy, Thieling, Gillespie, & Smith, 2008; Stranko et al., 2008), as well as increased water temperatures (Bassar, Letcher, Nislow, & Whiteley, 2016; Chadwick, Nislow, & McCormick, 2015; Stitt et al., 2014; Warren, Robinson, Josephson, Sheldon, & Kraft, 2012), and non‐native species (Wagner, Deweber, Detar, & Sweka, 2013). Although the plight of Brook Trout has sparked considerable interest and increased research in recent years, many regions still lack sufficient information to accurately assess the status of Brook Trout at the population level (Hudy et al., 2008). Biological assessments and surveying efforts are crucial to identify populations in need of management, to protect healthy populations, and to monitor population trends.

Incorporating genetic tools into existing biological surveys can provide key insight into the status of populations. Small, isolated populations are at increased risk of genetic diversity loss and inbreeding depression, leading to a greater potential for extirpation (Frankham, Ballou, & Briscoe, 2009). In many fish species including Brook Trout, population fragmentation, and the subsequent loss of genetic diversity, can occur via physical barriers to fish movement, whether due to natural (waterfalls) or man‐made (dams and culverts) sources (Nathan, Smith, Welsh, & Vokoun, 2018; Timm, Hallerman, Dolloff, Hudy, & Kolka, 2016; Torterotot, Perrier, Bergeron, & Bernatchez, 2014; Whiteley et al., 2013). Isolation can also result from the extirpation of neighboring populations (Letcher, Nislow, Coombs, O'Donnell, & Dubreuil, 2007), as well as from thermal barriers, when high temperatures prevent migration between populations (Aunins, Petty, King, Schilz, & Mazik, 2015). Therefore, maintaining and restoring population connectivity are critical to the successful management of Brook Trout populations. Genetic tools can aid in identifying at‐risk populations by providing information on the genetic diversity, effective population size, and level of gene flow among populations.

Due to their popularity in the recreational fishing industry, hatchery‐reared Brook Trout are frequently stocked into streams and lakes. Although stocking can offset some of the angling pressure caused by recreational fishing (Askey, Parkinson, & Post, 2013), there can be unintended consequences of this practice. Hatchery introgression, occurring when hatchery fish spawn with wild individuals, can result in a long‐term loss of genetic diversity in the wild population as well as a reduction in the genetic differentiation among populations (Eldridge, Myers, & Naish, 2009; Lamaze, Sauvage, Marie, Garant, & Bernatchez, 2012; Marie, Bernatchez, & Garant, 2010; Perrier, Guyomard, Bagliniere, Nikolic, & Evanno, 2013; Valiquette, Perrier, Thibault, & Bernatchez, 2014). However, the degree to which hatchery introgression occurs can vary substantially. For Brook Trout, studies assessing stocked lakes have found moderate‐to‐high levels of hatchery introgression (Harbicht, Alshamlih, Wilson, & Fraser, 2014; Lamaze et al., 2012; Létourneau et al., 2017; Marie et al., 2010), whereas introgression in small stream systems is often limited (Bruce & Wright, 2018; Kelson, Kapuscinski, Timmins, & Ardren, 2015; White, Miller, Dowell, Bartron, & Wagner, 2018). This trend could be due to greater dispersal ability for Brook Trout stocked into streams, as fish can more easily move into neighboring tributaries, rather than remain confined to a single lake. Marie, Bernatchez, and Garant (2012) found a negative relationship between hatchery introgression and lake surface area, suggesting that the amount of available habitat influences the level of wild‐hatchery introgression. Therefore, a connected stream network may represent a larger amount of Brook Trout habitat than a single lake, decreasing the frequency of wild‐hatchery encounters and the frequency of introgression.

In New York State, large portions of stream habitat require increased monitoring in order to effectively manage stream populations of Brook Trout. The Eastern Brook Trout Joint Venture (2016) found that only 10% of subwatersheds in New York State contained intact Brook Trout populations, while a majority of the state's waters have been extirpated (43%) or have lost a large portion of the Brook Trout habitat (47%), defined as habitat capable of maintaining self‐sustaining Brook Trout populations. With fewer neighboring populations to exchange migrants, the remaining Brook Trout populations likely exhibit greater isolation, increasing the likelihood of additional extirpation events, and even basin‐wide extinctions (Letcher et al., 2007). Historic widespread stocking of hatchery Brook Trout has led many to question the genetic integrity of the present‐day populations in New York State (Perkins, Krueger, & May, 1993); however, this has yet to be empirically examined over a broad geographic range.

The primary purpose of this study was to provide quantitative data on Brook Trout populations throughout western New York State that can aid in conservation management. To accomplish this goal, our study examined 75 wild Brook Trout populations, spanning the Allegheny, Erie/Niagara, Genesee, and Susquehanna drainage basins, as well as portions of the Oswego and Lake Ontario basins. The main objectives were to (a) characterize the genetic diversity, relatedness, and effective population sizes within Brook Trout populations; (b) quantify the degree of connectivity among the populations; and (c) evaluate the level of hatchery introgression occurring in wild, stream‐dwelling populations of Brook Trout.

## 2 METHODS

### 2.1 Sample collection

**Figure 1:**
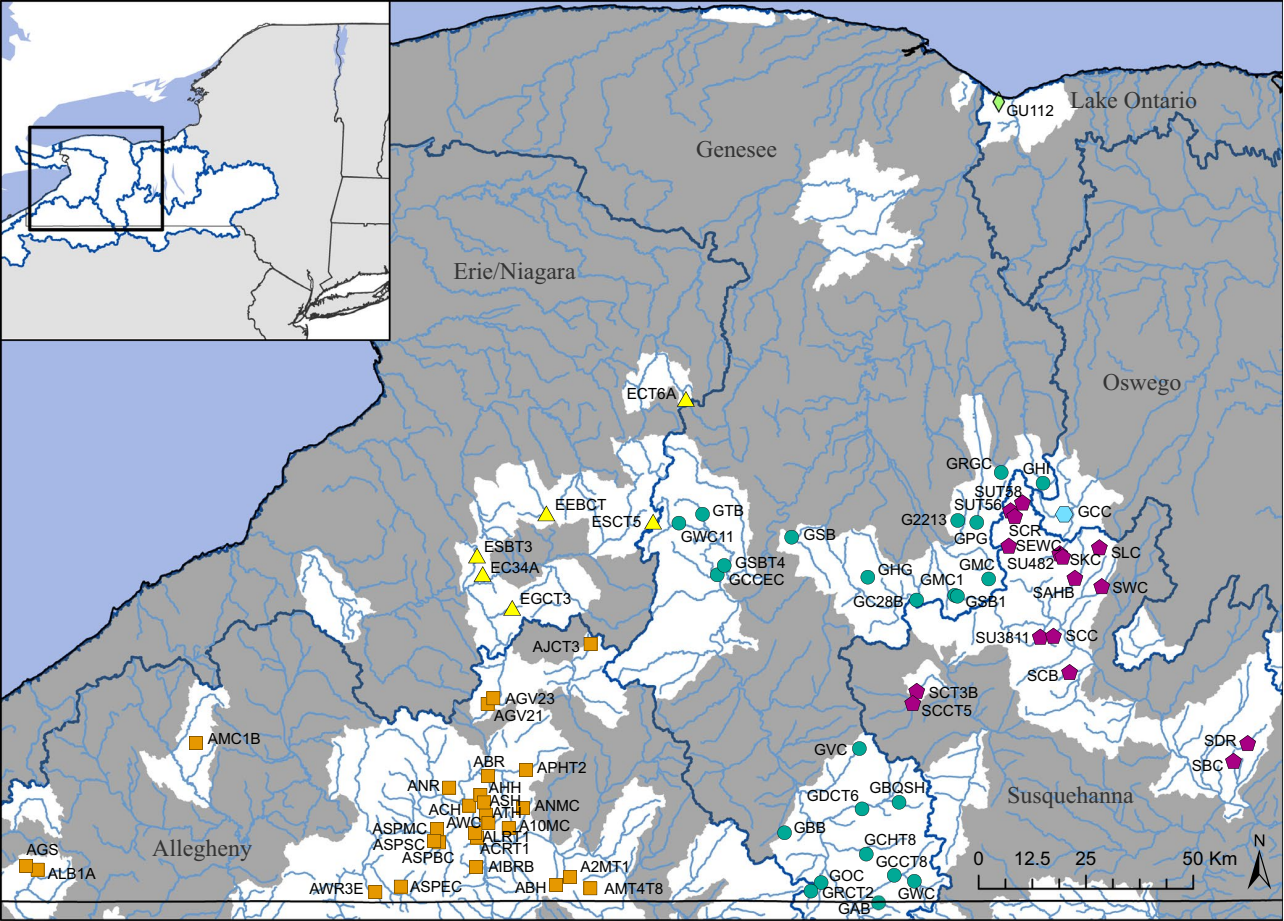
Map of New York State showing the 75 wild Brook Trout sampling localities. Major drainage basins are delineated and labeled. Sample localities within separate drainage basins are represented by different colors and symbols. Subwatersheds (HUC 12) where Brook Trout have been extirpated, based on the Eastern Brook Trout Joint Venture (2016) report, are shown in gray

A total of 3,436 fin clips were collected from wild Brook Trout by the New York State Department of Environmental Conservation (NYSDEC) and the USFWS Lower Great Lakes Fish and Wildlife Conservation Office. The collected fin clips represented a mixed‐age sample of each population, preferentially including individuals of age one year or older to avoid biasing the diversity and relatedness calculations. Sampling took place at 75 sites, all located in stream habitats, distributed throughout six major drainage basins, including the Allegheny (27 sites), Erie/Niagara (6 sites), Genesee (24 sites), Susquehanna (16 sites), Oswego (1 site), and Lake Ontario (1 site) drainage basins (Figure 1, Table 1). For clarity, throughout this paper we refer to 6‐digit hydrologic unit code (HUC) drainages as basins, 8‐digit HUC drainages as subbasins, 10‐digit HUC drainages as watersheds, and 12‐digit HUC drainages as subwatersheds.

**Table 1:** Locality information for Brook Trout sampled in western New York State. Watershed subdivisions for 8‐digit, 10‐digit, and 12‐digit hydrologic unit codes (HUC) are provided

HUC 8	HUC 10	HUC 12	Stream	ID	*N*_C_	*N_G_*
Allegheny basin						
Conewango	Cassadaga Ck	Mill Ck (1)	Mill Ck, T−1B	AMC1B	7	7
Upper Allegheny	Brokenstraw Ck	Brownell Branch‐Brokenstraw Ck (2)	Gallop (Town) Stream	AGS	8	8
			Little Brownell, T−1A	ALB1A	48	48
	Ischua Ck	Saunders Ck (3)	Johnson Ck, T−3	AJCT3	50	50
	Great Valley Ck	Upper Great Valley Ck (4)	Great Valley Ck, T−23	AGV23	48	48
			Great Valley Ck, T−21	AGV21	13	13
		Wrights Ck (5)	Pumpkin Hollow and T−2	APHT2	49	49
			Barker Run	ABR	50	50
	HW Allegheny R	Indian Ck (6)	Mix Ck	AMT4T8	50	50
	Upper Allegheny R	Fourmile Ck (7)	Twomile Ck and T−1	A2MT1	50	50
			Bucher Hollow	ABH	31	30
		Chipmunk Ck (8)	Nine Mile Ck	ANMC	50	50
			Ten Mile Ck	A10MC	50	50
	Middle Allegheny R	Bucktooth Run (9)	Newton Run	ANR	50	50
		Windfall Ck (10)	Christian Hollow	ACH	50	49
			Hardscrabble Hollow	AHH	50	50
			Sullivan Hollow	ASH	50	49
			Thorpe Hollow	ATH	50	50
			Windfall Ck	AWC	50	50
			Leonard Run and T−1	ALRT1	50	49
			Carrollton Run and T−1	ACRT1	47	47
		Red House Brook (11)	McIntosh Ck	ASPMC	50	50
			Stoddard Ck	ASPSC	50	50
			Beehunter Ck	ASPBC	50	50
	Lower Allegheny R	Wolf Run (12)	Wolf Run and T−3	AWR3E	56	55
		Quaker Run (13)	English Ck	ASPEC	50	50
	Tunungwant Ck	Outlet Tunungwant Ck (14)	Irish and Rice Brooks	AIBRB	50	50
Erie/Niagara basin						
Cattaraugus	HW Cattaraugus Ck	Spring Brook‐Cattaraugus Ck (1)	Spring Brook and T−3	ESBT3	50	50
			Cattaraugus Ck, T−34A	EC34A	50	50
		Buttermilk Ck (2)	Gooseneck Ck and T−3	EGCT3	50	50
		HW Cattaraugus Ck (3)	Spring Ck off West Hill Rd	ESCT5	49	48
Niagara	Buffalo R	HW E Branch Cazenovia Ck (4)	East Branch Cazenovia Ck	EEBCT	51	51
Buffalo‐Eighteenmile	Upper Tonawanda Cr	Crow Ck (5)	Crow Ck	ECT6A	50	50
Genesee basin						
Upper Genesee	HW Genesee R	HW Genesee R (1)	Ainsworth Brook	GAB	51	51
	Cryder Ck/Genesee R	Marsh Ck (2)	Redwater Ck and T−2	GRCT2	50	50
			Orebed Ck	GOC	50	50
		Chenunda Ck (3)	Chenunda‐T8 Ck	GCHT8	33	33
		Cryder Ck (4)	Cryder T8	GCCT8	50	49
		Marsh Ck (5)	Wileyville Ck	GWC	48	48
	Dyke Ck	Middle Dyke Ck (6)	Dyke Ck, T−6	GDCT6	15	15
		Upper Dyke Ck (7)	Best, Quig, Shovel Hollow	GBQSH	50	50
	Van Campen Ck/Genesee R	Brimmer Brook (8)	Brimmer Brook	GBB	50	50
		Vandermark Ck (9)	Vandermark Ck	GVC	50	48
	Cold Ck/Genesee R	Cold Ck (10)	Cold Ck and Elm Ck	GCCEC	50	50
	Wiscoy Ck	Wiscoy Ck (11)	Spencer Brook T−4 of Wiscoy	GSBT4	50	50
		HW Wiscoy Ck (12)	Wiscoy Ck T−11	GWC11	50	50
		Trout Brook (13)	Trout Brook and Tribs	GTB	50	50
	Canaseraga Ck	HW Keshequa Ck (14)	Spring Brook	GSB	40	40
		HW Canaseraga Ck (15)	Hovey Gully	GHG	50	50
		Bennett Ck (16)	Canaseraga Ck, T−28B	GC28B	50	50
		Stony Brook (17)	Stony Brook	GSB1	50	50
			Mill Ck	GMC1	50	50
		Mill Ck (18)	Mill Ck	GMC	50	50
			Unnamed trib	G2213	40	40
Lower Genesee	HW Honeoye Ck	Hemlock Lake (19)	Pokamoonshine Gulf	GPG	50	50
			Reynolds Gully Ck	GRGC	50	48
		Honeoye Inlet (20)	Honeoye Inlet	GHI	32	32
Oswego basin						
Seneca	Canandaigua Lake	Naples Ck (21)	Grimes Ck	GCC	50	49
Lake Ontario basin						
Irondequoit‐Ninemile	Irondequoit Ck‐Frontal Lake Ontario	West Ck‐Frontal Lake Ontario (22)	Unnamed trib	GU112	20	20
Susquehanna basin						
Tioga	Canacadea Ck	Upper Canacadea Ck (1)	Canacadea Ck, T−5	SCCT5	50	50
		Lower Canacadea Ck (2)	Canacadea Ck, T−3B	SCT3B	38	36
Chemung	Upper Cohocton R	Punky Hollow (3)	Unnamed trib	SUT58	50	50
			Unnamed trib	SUT56	46	46
			Cohocton R	SCR	50	49
			East Wayland Ck	SEWC	50	50
		Reynolds Ck (4)	Unnamed trib	SU482	47	47
			Kirkwood Ck	SKC	42	42
		Twelvemile Ck (5)	Lyon Ck	SLC	46	46
			Avery Hollow Brook	SAHB	50	50
		Tenmile Ck (6)	West Ck	SWC	50	49
			Cotton Ck	SCC	50	49
		Neils Ck (7)	Unnamed trib	SU3811	50	49
	Middle Cohocton R	Campbell Ck (8)	Chamberlain Brook	SCB	50	49
	Lower Cohocton R	Dry Run (9)	Dry Run	SDR	50	50
	Upper Chemung R	Cutler Ck (10)	Borden Ck	SBC	31	31
Hatchery						
			Randolph Fish Hatchery—Rome strain	ROM	50	50
			Oswayo Hatchery—Oswayo strain	OSW	50	50
			Oswayo Hatchery—Tylersville strain	TYL	50	50

Note

Numbers in parentheses correspond to the HUC 12 subwatershed delineations in Figure 6.

Abbreviations: Ck, creek; HW, headwater; *N_C_*, sample size collected; *N_G_*, sample size genotyped; R, river.

Brook Trout stocked by the NYSDEC in western New York State are exclusive of the Rome strain reared at the Randolph State Fish Hatchery. Although approximately 55% of the study streams have been directly stocked in the past, none are currently being stocked with hatchery Brook Trout. The NYSDEC has not stocked hatchery Brook Trout in streams occupied by wild Brook Trout in the study area in over ten years, and many of the study sites have not been directly stocked since the 1970s (mean last year stocked: 1954; Table A1 in Appendix S1 ). However, stocking has occurred more recently in tributaries adjacent to some streams in our study, and at a broader scale, Brook Trout stocking continues in two watersheds within our study region.

The Oswayo State Fish Hatchery, operated by the Pennsylvania Fish and Boat Commission, stocks Brook Trout in Pennsylvania counties bordering New York State that could potentially come in contact with our study sites near the New York/Pennsylvania border, located in the Allegheny and Genesee basins. In order to detect hatchery individuals and hatchery introgression in wild Brook Trout populations, samples were collected from the Rome strain (*N* = 50), as well as from the two Brook Trout strains stocked by the Oswayo State Fish Hatchery, the Oswayo strain (*N* = 50), and the Tylersville strain (*N* = 50).

### 2.2 Laboratory methods

Genomic DNA was extracted using the Mag‐Bind ® Tissue DNA Kit (Omega Bio‐tek) with the KingFisher Flex Magnetic Particle Processor (Thermo Fisher Scientific) as well as the Puregene (Gentra Systems, Inc.) methods, following the manufacturers’ protocols. Samples were genotyped at 12 microsatellite markers developed in Brook Trout: *SfoB52*, *SfoC38*, *SfoC113*, *SfoD75*, *SfoD100*, *SfoC28*, *SfoC86*, *SfoC88*, *SfoC129*, *SfoC24*, *SfoC115*, and *SfoD91* (King, Lubinski, Burnham‐Curtis, Stott, & Morgan, 2012). Markers were combined into three multiplex reactions for PCR amplification and fragment analysis. Each 15 µl PCR consisted of 1.5 µl genomic DNA extract, 1.5× PCR buffer, 3.75 mM MgCl_2_, 0.3175 mM dNTPs, 0.08–0.18 µM of each primer, and 0.08 units/µl GoTaq® Flexi DNA polymerase (Promega). The amplification protocol followed that of King et al. (2012). PCR products were then visualized on an ABI 3130XL genetic analyzer (Life Technologies), and alleles were scored with GeneMapper 5 (Life Technologies) by two independent readers. As a quality control measure, 10% of the samples were re‐extracted and genotyped to compare against the original data.

### 2.3 Genetic diversity within populations

Because some populations were sampled during multiple field surveys, we performed an identity test in Cervus 3.0.7 (Kalinowski, Taper, & Marshall, 2007) to identify individuals that had been sampled more than once. For individuals with matching alleles across all genotyped loci, one individual from the pair was removed from the data set. Deviations from Hardy–Weinberg equilibrium and linkage equilibrium were assessed using Fisher exact tests with 3,200 iterations in GDA 1.1 (Lewis & Zaykin, 2001). Significance was assessed after applying a Bonferroni correction to account for multiple comparisons (Rice, 1989).

Estimates of genetic diversity, including average number of alleles per locus (*A*), observed and expected heterozygosity (*H*_o_ and *H*_e_ *_,_* respectively), and the inbreeding coefficient (*f*), were calculated with GDA 1.1 (Lewis & Zaykin, 2001), while allelic richness (*A*_r_) was calculated with FSTAT 2.9.3 (Goudet, 1995). Populations with fewer than 30 individuals were removed from within‐population genetic diversity calculations to avoid biasing the interpretation. We calculated effective population sizes (*N*_e_) for all wild populations and effective number of breeders (*N*_b_) for the single‐cohort samples of hatchery strains in NeEstimator 2.01 (Do et al., 2014). The linkage disequilibrium (LD) method was used, excluding alleles at frequencies below 0.02 to minimize inflated estimates caused by rare alleles (Waples & Do, 2010). We used the jackknife method to calculate 95% confidence intervals, which has been shown to perform as well or better than the parametric method (Waples & Do, 2010). To examine whether *N*_e_ estimates show a correlation with *A*_R_, as one would expect if they relate to recent genetic drift (Wright, 1931), we calculated Pearson's correlation coefficient in R (R Core Team, 2014).

To determine how closely related individuals within a given sampling location were to each other, we calculated maximum‐likelihood estimates of relatedness (*r*) with ML‐Relate (Kalinowski, Wagner, & Taper, 2006). This method has been shown to produce lower error rates than other relatedness estimates (Milligan, 2003). Typically, relatedness values of 0.5 indicate a full‐sibling or parent–offspring relationship and values of 0.25 indicate a half‐sibling relationship. From the resulting pairwise relatedness values, the average level of relatedness was calculated for each site. Additionally, the standard relatedness values of 0.5 and 0.25 were used as minimum cutoff values to pool pairwise comparisons into categories (*r* ≥ 0.5 and 0.25 ≤ *r* < 0.5) to determine the proportion of the population with family‐level relatedness values. As a comparison, maximum‐likelihood estimates of relationship (parent–offspring, full‐sibling, half‐sibling, and unrelated) were determined between all pairs of individuals within each population using ML‐Relate. Line graphs of genetic diversity estimates, effective population size, and average relatedness were then generated with the ggplot2 package in R v.3.4.1 (R Core Team, 2014; Wickham, 2009) to visualize diversity trends across all populations examined.

Differences in genetic diversity metrics among the four major basins (Allegheny, Erie/Niagara, Genesee, and Susquehanna) were assessed with the Kruskal–Wallis and pairwise Wilcoxon rank‐sum tests in R (R Core Team, 2014). To understand the influence of isolation on genetic diversity, we examined the relationship between each of the diversity metrics compared to population density, defined as the number of populations sampled within the same subwatershed. While every effort was made to acquire genetic samples from all existing Brook Trout populations in the region, this was not possible for some due to inaccessibility or inability to collect a sufficient sample size. However, the number of sampled populations likely approximates the number of Brook Trout populations available for migrant and allele exchange. Because many subwatersheds contained multiple populations, we created an R script to minimize the autocorrelation effects associated with concurrently analyzing the same subwatershed more than once. We performed 1,000 bootstrap replicates, each time randomly selecting one population from each subwatershed, along with the associated diversity metric and Brook Trout population density for that subwatershed. A bootstrap correlation coefficient was then calculated using Spearman's rank‐order correlation to determine statistical significance.

### 2.4 Genetic differentiation among populations

Pairwise *F*_ST_ values were calculated across all sites with Arlequin 3.5 (Excoffier & Lischer, 2010) to examine the level of genetic differentiation among Brook Trout sampling localities. Significance was assessed with 10,000 permutations and based on a Bonferroni‐adjusted *p*‐value (Rice, 1989). To further visualize the relationships among Brook Trout localities, we performed a principal coordinate analysis (PCoA) in GenAlEx 6.5 (Peakall & Smouse, 2012) using the previously calculated *F*_ST_ values. The R package ggplot2 (Wickham, 2009) was used to graph the resulting PCoA, as well as to graphically display the pairwise *F*_ST_ values in a heat map matrix. Differences in within‐basin *F*_ST_ values were examined across the four major basins with the Kruskal–Wallis and pairwise Wilcoxon rank‐sum test in R (R Core Team, 2014).

A hierarchical analysis of molecular variance (AMOVA) was carried out in Arlequin 3.5 (Excoffier & Lischer, 2010), and statistical significance was assessed with 10,000 permutations. Sampling sites were grouped based on HUC 6, HUC 8, HUC 10, and HUC 12 drainages to examine whether the genetic variation within the data set could be explained by higher‐order hydrologic subdivisions. Genetic partitioning among Brook Trout sample localities was additionally evaluated with the individual‐based assignment test in GeneClass2 (Piry et al., 2004) using the Bayesian method by Rannala and Mountain (1997). This analysis tests how well each individual can be genetically assigned to its collection locality, with highly differentiated populations exhibiting greater proportions of correct assignment.

We performed an isolation‐by‐distance (IBD) analysis to determine whether geographic distance was influencing the patterns of genetic differentiation among Brook Trout populations. Pairwise river distances were calculated using the R package RIVERDIST (Tyers, 2016). We converted the previously calculated *F*_ST_ values into the Rousset (1997) linearized *F*_ST_ metric and carried out a Mantel test with the R package ape (Paradis & Schliep, 2018).

### 2.5 Population admixture and hatchery introgression

To examine the degree of admixture among Brook Trout populations throughout western New York, we performed a Bayesian clustering analysis with STRUCTURE 2.3.4 (Pritchard, Stephens, & Donnelly, 2000). Sampling localities within each major drainage basin were analyzed together, along with the Rome strain, in order to determine the degree of hatchery introgression within wild populations. Because the Allegheny and Genesee basins drain regions that extend into Pennsylvania and contain sites near the New York/Pennsylvania border, the Oswayo and Tylersville Brook Trout strains were also included in the analysis for these two basins.

The default parameters (correlated allele frequency model with a uniform prior distribution for alpha) were applied with run lengths of 100,000 iterations as burn‐in and 500,000 additional iterations. An alternative parameter set was also applied for run lengths of 100,000 iterations as burn‐in and 250,000 iterations after the burn‐in. The alternative parameter set consisted of the independent allele frequency model, inferring alpha independently for each population, and setting the initial alpha prior to ~1/*K*. This combination of parameters was recommended by Wang (2017) for data sets with many populations and unbalanced sampling across populations and has been shown to improve the accuracy of the clustering results. The range of genetic clusters (*K*) examined varied based on the number of sampling sites within a given drainage basin (Allegheny: *K* = 1–31, Erie/Niagara: *K* = 1–8, Genesee (included sites GU112 and GCC): *K* = 1–30, Susquehanna: *K* = 1–18), and 10 replicates were performed for each *K* value.

To minimize the erroneous effects of IBD on population clustering by STRUCTURE (Frantz, Cellina, Krier, Schley, & Burke, 2009; Schwartz & McKelvey, 2009), population‐specific analyses were also performed where each wild population was examined individually along with the appropriate hatchery strain(s), applying both the default and alternative Wang (2017) parameter sets. Populations in the Erie/Niagara and Susquehanna were examined with only the Rome strain (*K* = 1–3), and populations within the Allegheny, Genesee, Lake Ontario, and Oswego basins were examined with all three hatchery strains (*K* = 1–5). Ten runs were performed for each *K* value with an initial burn‐in of 250,000 iterations and an additional 500,000 iterations after the burn‐in.

The optimal *K* value for each analysis was inferred by examining both the mean log probability of the data (Pritchard et al., 2000) as well as by calculating Δ*K* (Evanno, Regnaut, & Goudet, 2005) with the web‐based program STRUCTURE HARVESTER (Earl & vanHoldt, 2012). To minimize unlikely clustering patterns, five runs with the highest likelihood scores were combined with CLUMPP 1.1.2 (Jakobsson & Rosenberg, 2007) and plotted with distruct 1.1 (Rosenberg, 2004). Potential wild‐hatchery introgression was identified based on the proportion of membership (*q*) to a genetic cluster associated with a hatchery strain, with *q* ≥ 0.10 used as a minimum. Prior studies have determined this threshold *q*‐value to produce the highest proportion of correctly assigned pure and hybrid individuals (Vähä & Primmer, 2006) and have been previously used to examine hatchery introgression in Brook Trout (Harbicht et al., 2014; Humston et al., 2012).

To better understand not only the degree of hatchery introgression in wild Brook Trout populations, but also the possible explanations for our results, we explored the influence of time on the levels of hatchery introgression in our study populations. Specifically, we examined the relationship between the mean membership proportions (*q*‐values) to the Rome hatchery strain and the number of years since the population was stocked by the NYDEC, either directly or via stocking an adjacent stream, using the most recent stocking year. A linear regression analysis was performed in R (R Core Team 2014) to evaluate the trend and significance of this relationship. Lastly, we examined the length of time in generations that backcrossing with a wild population would reduce the genetic signal of hatchery introgression. Using HYBRIDLAB 1.0 (Nielsen, Bach, & Kotlicki, 2006), we simulated random matings between a wild population (ATH, *N* = 50) and a hatchery population (Rome, *N* = 50), producing genotypic profiles for 50 wild‐hatchery hybrids. Ten generations of backcrossing with the wild population were then simulated, with 50 offspring produced for each generation. The resulting genotype data were then analyzed with STRUCTURE, *K* = 2, following the same methods as the population‐specific analyses described above.

## 3 RESULTS

Based on the 10% quality assessment, the genotyping error rate was determined to be 0.002 (nine single‐locus genotype mismatches were detected out of the 379 individuals re‐examined at 12 loci). Four individuals (AWR3E‐06, GCCT8‐47, SU3811‐36, and SCC‐35) failed to amplify and were removed from the data set, while individuals retained for further analysis displayed missing data at five or fewer loci. The identity test detected 15 sample pairs with exact allele matches across all genotyped loci. A majority of these duplicates originated from separate sampling events at the same locality. One matching pair (ACH‐38 and AHH‐42) was sampled from neighboring tributaries during separate survey dates, suggesting that Brook Trout individuals are capable of moving between adjacent tributaries. One individual from each of the 15 matching pairs was subsequently removed from the data set.

There were 10 instances of loci showing significant departures from Hardy–Weinberg expectations after applying the Bonferroni‐adjusted *p*‐value of 0.00005. Significant disequilibrium occurred for locus *SfoC28* in two populations (ASH and GCCT8), locus *SfoD91* in two populations (GSB1 and GCHT8), and loci *SfoD75*, *SfoC115*, *SfoD100*, *SfoC88*, *SfoB52*, and *SfoC113* in a single population each (AHH, GCHT8, GCCT8, EC34A, GCCEC, and GRGC, respectively). The observed deviations were not consistent across populations, indicating that null alleles were not likely the cause of the disequilibrium, and therefore, all loci were retained for the remainder of the analyses.

After removing 19 sampled individuals from the data set, a total of 3,417 wild and 150 hatchery Brook Trout individuals amplified consistently and displayed unique genotypes when all 12 markers were examined (Table 1). Overall, 1,202 individuals were analyzed from the Allegheny basin, 299 from the Erie/Niagara basin, 1,104 from the Genesee basin, 49 from the Oswego basin, 20 from the Lake Ontario basin, 743 from the Susquehanna basin, and 50 from each of the three hatchery strains.

### 3.1 Genetic diversity within populations

**Figure 2:**
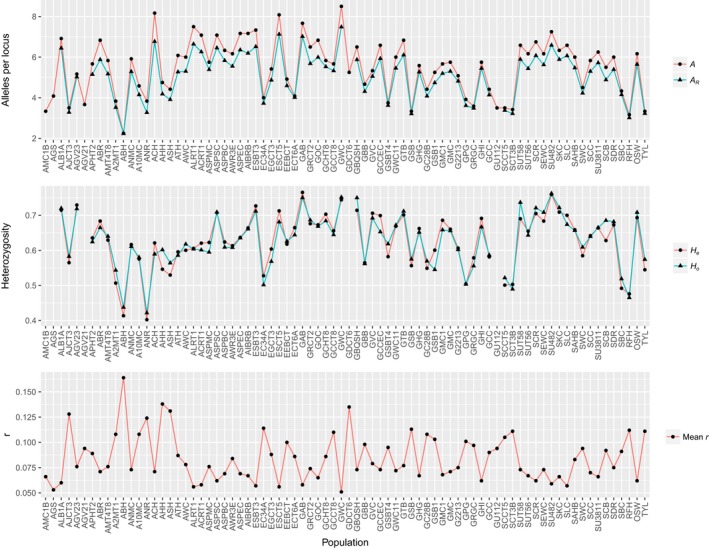
Line graphs showing genetic diversity estimates for Brook Trout populations in western New York State. Top: the average number of alleles per locus (*A*) and allelic richness (*A*_R_); Middle: expected and observed heterozygosity (*H*_e_ and *H*_o_, respectively); Bottom: mean maximum‐likelihood estimate of pairwise relatedness (*r*)

Genetic diversity estimates showed a large degree of variation among localities (Figure 2, Table 2). Across all wild Brook Trout populations examined, the mean *A*_R _was found to be 5.148 (2.23–7.485, standardized to a minimum sample size of 30), and mean *H*_e_ and *H*_o_ were 0.630 (0.402–0.766) and 0.630 (0.437–0.752), respectively. There were 10 sites showing *A* and *A*_R_ values of less than four alleles per locus (of sites with *N* > 30), including AJCT3, A2MT1, ABH, ANR, GSBT4, GSB, GPG, GRGC, SCCT5, and SCT3B. The ROM and TYL hatchery strains were also found to have *A* and *A*_R_ estimates below 4 alleles per locus. In contrast, 11 sites exhibited over 7 alleles per locus for estimates of *A*: ACH, ALRT1, ACRT1, ASPSC, ASPEC, AIBRB, ESBT3, ESCT5, GAB, GWC, and SU482. Populations GAB, GWC, GBQSH, and SU482 all had *H*_o_ values above 0.75, while populations ABH and ANR were found to have the lowest *H*_o_ (<0.45). Inbreeding coefficient values (*f*) ranged from −0.103 in population AHH, suggesting heterozygote excess which is common in small populations (Allendorf, Luikart, & Aitken, 2013), to 0.093 in population GSB1.

**Table 2:** Genetic diversity estimates for Brook Trout populations in western New York State, including sample size (*N*), mean number of alleles per locus (*A*), allelic richness based on *N* = 30 (*A*_r_), expected heterozygosity (*H*_e_), observed heterozygosity (*H*_o_), and inbreeding coefficient (*f*). Effective population size estimates (*N*_e_) are provided with 95% confidence intervals (CI) based on the jackknife method. Mean estimates for each major drainage basin are in bold

Pop ID	*N*	*A*	*A*_r_	*H*_e_	*H*_o_	*f*	*N*_e_ (95% CI)
Allegheny basin							
AMC1B	7	3.333	*–*	*–*	*–*	*–*	*–*
AGS	8	4.083	*–*	*–*	*–*	*–*	*–*
ALB1A	47.5	6.917	6.441	0.715	0.720	−0.007	61.1 (44.5–91.3)
AJCT3	49.7	3.500	3.284	0.565	0.583	−0.032	14.0 (8.9–21.7)
AGV23	48	5.167	5.026	0.729	0.719	0.015	28.4 (21.9–37.8)
AGV21	13	3.667	*–*	*–*	*–*	*–*	*–*
APHT2	49	5.667	5.149	0.636	0.624	0.018	17.7 (13.4–23.6)
ABR	49.9	6.833	5.878	0.684	0.665	0.028	35.7 (26.2–51.4)
AMT4T8	50	5.833	5.175	0.629	0.640	−0.017	30.6 (20.8–48.5)
A2MT1	50	3.833	3.523	0.507	0.543	−0.073	21.0 (12.7–37.0)
ABH	29.8	2.250	2.231	0.414	0.437	−0.059	4.8 (2.1–11.9)
ANMC	49.9	5.917	5.289	0.617	0.611	0.009	40.9 (30.5–57.8)
A10MC	50	4.583	4.146	0.576	0.580	−0.008	11.5 (7.9–16.3)
ANR	50	3.833	3.272	0.402	0.422	−0.049	30.4 (18.9–54.6)
ACH	49.8	8.167	6.770	0.621	0.589	0.052	33.1 (26.8–41.7)
AHH	48.1	4.750	4.185	0.546	0.602	−0.103	8.4 (5.3–11.8)
ASH	46.8	4.417	3.911	0.530	0.564	−0.065	4.1 (2.9–9.3)
ATH	48.6	6.083	5.272	0.596	0.586	0.017	21.0 (15.3–29.7)
AWC	49.5	6.000	5.304	0.600	0.618	−0.029	30.0 (21.8–43.4)
ALRT1	49	7.500	6.644	0.606	0.604	0.003	55.6 (40.3–83.0)
ACRT1	47	7.083	6.262	0.621	0.601	0.032	72.0 (47.2–131.6)
ASPMC	49.9	5.750	5.385	0.623	0.595	0.045	26.9 (20.5–36.4)
ASPSC	49.7	7.083	6.455	0.708	0.709	−0.002	50.7 (38.1–71.5)
ASPBC	49.7	6.333	5.831	0.624	0.609	0.024	31.5 (25.3–40.1)
AWR3E	53.6	6.167	5.551	0.613	0.608	0.009	19.6 (13.3–29.8)
ASPEC	49.6	7.167	6.355	0.637	0.635	0.002	27.7 (21.4–36.8)
AIBRB	49.5	7.167	6.192	0.664	0.661	0.005	50.8 (37.9–72.5)
		**5.522**	**5.147**	**0.603**	**0.605**	**−0.008**	**30.3**
Erie/Niagara basin							
ESBT3	50	7.333	6.516	0.727	0.712	0.021	105.7 (65.4–229.5)
EC34A	50	4.000	3.726	0.528	0.502	0.050	10.8 (7.9–14.5)
EGCT3	50	5.417	4.869	0.604	0.568	0.059	16.8 (12.8–22.2)
ESCT5	47.8	8.083	7.126	0.713	0.681	0.045	83.0 (61.0–123.4)
EEBCT	51	4.917	4.591	0.618	0.626	−0.013	13.0 (9.9–17.0)
ECT6A	49.8	4.083	4.014	0.665	0.644	0.032	37.5 (25.4–60.6)
		**5.639**	**5.141**	**0.642**	**0.622**	**0.032**	**44.5**
Genesee basin							
GAB	50.6	7.667	7.016	0.766	0.750	0.020	50.2 (36.6–74.0)
GRCT2	49.9	6.500	5.681	0.676	0.686	−0.015	32.2 (24.6–43.7)
GOC	50	6.833	5.993	0.673	0.668	0.007	66.9 (47.0–106.1)
GCHT8	33	5.833	5.527	0.703	0.684	0.027	12.1 (9.4–15.7)
GCCT8	49	5.667	5.327	0.656	0.645	0.018	3.8 (3.3–5.0)
GWC	48	8.500	7.485	0.744	0.752	−0.010	70.5 (50.0–110.8)
GDCT6	15	5.250	*–*	*–*	*–*	*–*	*–*
GBQSH	50	6.500	5.876	0.714	0.750	−0.051	48.6 (37.0–67.2)
GBB	50	4.667	4.308	0.565	0.562	0.005	10.4 (7.4–14.0)
GVC	47.9	5.333	5.054	0.706	0.692	0.020	28.4 (21.5–38.8)
GCCEC	49.7	6.583	5.929	0.699	0.653	0.067	26.2 (20.4–34.3)
GSBT4	50	3.750	3.622	0.582	0.618	−0.063	85.4 (37.3–1,751.7)
GWC11	50	6.000	5.456	0.669	0.672	−0.004	32.1 (21.7–51.4)
GTB	50	6.833	6.105	0.701	0.712	−0.015	15.9 (13.5–18.7)
GSB	40	3.333	3.210	0.556	0.575	−0.034	14.8 (9.4–23.8)
GHG	50	5.583	5.269	0.662	0.652	0.016	51.8 (35.2–85.6)
GC28B	49.7	4.417	4.081	0.549	0.569	−0.038	12.5 (9.9–15.8)
GSB1	50	5.250	4.737	0.601	0.545	0.093	12.8 (10.5–15.7)
GMC1	50	5.667	5.195	0.686	0.658	0.041	99.8 (54.0–338.2)
GMC	49.9	5.750	5.303	0.660	0.656	0.007	54.8 (34.1–107.3)
G2213	40	5.083	4.818	0.606	0.602	0.007	54.0 (34.5–102.8)
GPG	50	3.917	3.613	0.504	0.503	0.002	35.8 (22.3–65.6)
GRGC	48	3.583	3.485	0.579	0.556	0.041	39.4 (25.4–69.3)
GHI	32	5.750	5.448	0.691	0.667	0.036	60.5 (36.0–142.2)
		**5.594**	**5.154**	**0.650**	**0.645**	**0.008**	**40.0**
Oswego basin							
GCC	49	4.417	4.139	0.582	0.587	−0.009	38.3 (25.0–65.7)
Ontario basin							
GU112	20	3.500	*–*	*–*	*–*	*–*	*–*
Susquehanna basin							
SCCT5	50	3.500	3.379	0.501	0.522	−0.042	94.3 (39.8–Inf)
SCT3B	35.9	3.417	3.219	0.503	0.489	0.027	41.8 (19.3–208.0)
SUT58	50	6.583	5.891	0.690	0.737	−0.068	36.5 (28.9–47.6)
SUT56	46	6.167	5.437	0.655	0.643	0.018	51.2 (37.5–75.3)
SCR	49	6.750	6.076	0.705	0.721	−0.023	182.0 (89.5–1,921.7)
SEWC	50	6.167	5.623	0.684	0.708	−0.037	39.9 (30.1–55.5)
SU482	47	7.250	6.586	0.760	0.762	−0.004	87.0 (56.9–162.8)
SKC	41.6	6.333	5.885	0.709	0.722	−0.018	52.9 (36.8–84.7)
SLC	46	6.583	6.067	0.700	0.674	0.038	102.3 (60.0–262.2)
SAHB	50	6.000	5.473	0.659	0.657	0.003	23.0 (18.1–29.6)
SWC	49	4.500	4.227	0.585	0.609	−0.042	15.8 (12.0–21.0)
SCC	49	5.833	5.300	0.642	0.639	0.004	50.9 (36.6–76.7)
SU3811	49	6.250	5.721	0.664	0.665	−0.001	72.2 (50.1–117.9)
SCB	49	5.500	4.882	0.628	0.685	−0.092	19.6 (15.7–24.7)
SDR	50	6.000	5.392	0.673	0.682	−0.014	33.2 (25.3–45.1)
SBC	31	4.333	4.152	0.492	0.519	−0.056	43.1 (24.5–106.7)
		**5.698**	**5.207**	**0.641**	**0.652**	**−0.019**	**59.1**
Hatchery							
ROM	50	3.167	3.016	0.476	0.465	0.023	33.8 (17.4–87.2)^a^
OSW	50	6.167	5.643	0.693	0.708	−0.022	126.9 (76.2–309.2) a
TYL	50	3.333	3.225	0.545	0.574	−0.054	37.0 (23.8–65.0) a
		**4.222**	**3.961**	**0.571**	**0.582**	**−0.018**	**65.9**

a Effective number of breeders (*N*_b_).

The wild Brook Trout populations were comprised of mixed‐age samples, and therefore, *N*_e_ was calculated, while the hatchery strain samples consisted of a single cohort with calculations reflecting the number of breeders (*N*_b_). Estimates of *N*_e_ ranged from 3.8 (3.3–5.0) for site GCCT8 to 182.0 (89.5–1,921.7) for site SCR, with an overall mean of 41.4 across wild populations (Table 2, Figure A1 in Appendix S2). Our *N*_e_ estimates were low for many of the Brook Trout populations examined, with values below 10 for four populations, including GCCT8, ASH (*N*_e_ = 4.1; CI: 2.9–9.3), ABH (*N*_e_ = 4.8; CI: 2.1–11.9), and AHH (*N*_e_ = 8.4; CI: 5.3–11.8). In contrast, four populations exhibited estimates over 100, including SCR, ESBT3 (*N*_e_ = 105.7; CI: 65.4–229.5), SLC (*N*_e_ = 102.3; CI: 60.0–262.2), and the OSW hatchery strain (*N*_b_ = 126.9; CI: 76.2–309.2). Overall, we found a significant positive correlation between *N*_e_ and *A*_R_ (*r* = 0.399, *df* = 68, α = 0.05), suggesting that genetic drift, rather than sampling error, was largely responsible for the variation in diversity metrics across Brook Trout populations.

The maximum‐likelihood estimates of relationship for parent–offspring, full‐sibling, and half‐sibling were generally higher than estimates of relatedness (*r*) pooled into corresponding relationship categories, but showed similar trends across populations (Table 3). Overall, the relatedness estimates mirrored the genetic diversity results, where populations with low levels of diversity tended to have high levels of relatedness and vice versa (Figure 2, Table 3). For example, population ABH had the lowest *A* and *A*_R_ values, and displayed the highest mean *r* value (0.164). This population was also found to have the highest proportion of parent–offspring and full‐sibling relationships (0.126 and 0.113, respectively). Conversely, population GWC had the highest *A* and *A*_R_ values and exhibited the lowest mean *r* value (0.051).

**Table 3:** Maximum‐likelihood estimates of relatedness (*r*) and relationship for Brook Trout in western New York. Mean *r* values for each population are reported, as well as the proportion of pairwise comparisons with relatedness values ≥ 0.5 and 0.25 ≤ *r *< 0.5. The proportion of comparisons for relationship categories of parent–offspring (PO), full‐sibling (FS), and half‐sibling (HS) are also shown

Pop	Mean *r*	*r* ≥ 0.5	0.25 ≤ *r *< 0.5	PO	FS	HS
AMC1B	0.066	0.048	0.000	0.000	0.048	0.095
AGS	0.053	0.036	0.036	0.071	0.000	0.071
ALB1A	0.060	0.012	0.050	0.011	0.015	0.137
AJCT3	0.128	0.097	0.118	0.094	0.056	0.144
AGV23	0.076	0.012	0.086	0.006	0.033	0.161
AGV21	0.094	0.103	0.064	0.077	0.064	0.064
APHT2	0.089	0.054	0.081	0.040	0.043	0.113
ABR	0.071	0.026	0.064	0.023	0.024	0.128
AMT4T8	0.076	0.027	0.078	0.019	0.032	0.142
A2MT1	0.108	0.065	0.098	0.063	0.045	0.139
ABH	0.164	0.152	0.149	0.126	0.113	0.087
ANMC	0.073	0.026	0.068	0.022	0.021	0.135
A10MC	0.108	0.091	0.080	0.060	0.069	0.099
ANR	0.124	0.099	0.105	0.090	0.064	0.109
ACH	0.071	0.041	0.052	0.033	0.018	0.113
AHH	0.138	0.111	0.118	0.073	0.108	0.109
ASH	0.131	0.120	0.099	0.090	0.072	0.101
ATH	0.087	0.047	0.082	0.048	0.029	0.127
AWC	0.078	0.029	0.080	0.027	0.038	0.109
ALRT1	0.056	0.020	0.040	0.016	0.015	0.110
ACRT1	0.058	0.012	0.056	0.009	0.015	0.124
ASPMC	0.076	0.029	0.074	0.022	0.026	0.147
ASPSC	0.062	0.017	0.052	0.019	0.009	0.124
ASPBC	0.069	0.020	0.067	0.012	0.029	0.115
AWR3E	0.084	0.048	0.082	0.055	0.029	0.112
ASPEC	0.069	0.029	0.062	0.017	0.032	0.099
AIBRB	0.067	0.025	0.060	0.016	0.024	0.122
ESBT3	0.057	0.010	0.044	0.010	0.007	0.136
EC34A	0.114	0.091	0.087	0.072	0.058	0.133
EGCT3	0.088	0.046	0.079	0.038	0.037	0.133
ESCT5	0.056	0.008	0.039	0.009	0.007	0.132
EEBCT	0.100	0.056	0.096	0.049	0.042	0.160
ECT6A	0.086	0.028	0.080	0.024	0.024	0.164
GAB	0.058	0.013	0.045	0.009	0.016	0.113
GRCT2	0.074	0.030	0.063	0.027	0.024	0.128
GOC	0.065	0.021	0.051	0.016	0.018	0.118
GCHT8	0.086	0.042	0.095	0.023	0.057	0.108
GCCT8	0.110	0.094	0.097	0.054	0.077	0.128
GWC	0.051	0.011	0.035	0.004	0.015	0.105
GDCT6	0.135	0.133	0.086	0.038	0.133	0.105
GBQSH	0.073	0.029	0.057	0.016	0.036	0.113
GBB	0.098	0.069	0.080	0.053	0.044	0.134
GVC	0.079	0.035	0.078	0.024	0.027	0.148
GCCEC	0.073	0.017	0.077	0.016	0.028	0.137
GSBT4	0.095	0.033	0.105	0.048	0.037	0.139
GWC11	0.072	0.026	0.068	0.024	0.024	0.122
GTB	0.077	0.035	0.084	0.020	0.047	0.121
GSB	0.113	0.076	0.096	0.077	0.037	0.140
GHG	0.067	0.022	0.064	0.017	0.019	0.114
GC28B	0.108	0.081	0.088	0.073	0.054	0.111
GSB1	0.103	0.074	0.088	0.041	0.059	0.125
GMC1	0.068	0.016	0.048	0.016	0.013	0.148
GMC	0.071	0.020	0.060	0.016	0.012	0.145
G2213	0.075	0.017	0.090	0.017	0.026	0.138
GPG	0.101	0.058	0.104	0.056	0.039	0.149
GRGC	0.097	0.050	0.098	0.042	0.035	0.145
GHI	0.062	0.018	0.050	0.014	0.018	0.111
GCC	0.090	0.048	0.075	0.035	0.039	0.128
GU112	0.094	0.058	0.068	0.053	0.037	0.121
SCCT5	0.105	0.052	0.118	0.062	0.041	0.143
SCT3B	0.111	0.065	0.105	0.063	0.054	0.129
SUT58	0.073	0.028	0.060	0.019	0.029	0.117
SUT56	0.067	0.015	0.058	0.011	0.019	0.132
SCR	0.062	0.011	0.046	0.014	0.007	0.145
SEWC	0.073	0.026	0.060	0.014	0.031	0.125
SU482	0.059	0.012	0.040	0.009	0.013	0.125
SKC	0.066	0.019	0.067	0.016	0.023	0.114
SLC	0.057	0.011	0.034	0.009	0.012	0.127
SAHB	0.083	0.046	0.074	0.034	0.037	0.118
SWC	0.094	0.050	0.090	0.042	0.054	0.118
SCC	0.070	0.024	0.063	0.017	0.024	0.125
SU3811	0.066	0.008	0.073	0.009	0.014	0.153
SCB	0.092	0.108	0.123	0.027	0.072	0.111
SDR	0.075	0.032	0.063	0.029	0.029	0.127
SBC	0.091	0.052	0.084	0.049	0.045	0.120
ROM	0.112	0.064	0.109	0.065	0.041	0.152
OSW	0.062	0.011	0.051	0.011	0.012	0.133
TYL	0.111	0.069	0.105	0.066	0.042	0.137

Although we did not find statistical differences among the four major basins for any of the diversity metrics (Figure A2 in Appendix S2), overall differences in *N*_e_ were approaching significance (*p* = 0.060). Pairwise comparisons between the basins revealed that the *N*_e_ estimates for the Susquehanna basin were significantly higher than for the Allegheny (pairwise Wilcoxon rank‐sum test: *p* = 0.029).

Examining all drainage basins together, we found a weak, but positive relationship between population density, defined as the number of populations sampled within a subwatershed, and *A* that was approaching significance at an alpha level of 0.05 (mean *r* = 0.118, 95% CI: −0.012 to 0.245). For the Susquehanna basin alone, this relationship was statistically significant for all diversity metrics (*A*: mean *r *= 0.598, 95% CI: 0.477–0.749; *A*_R_: mean *r* = 0.611, 95% CI: 0.479–0.749; *H*_e_: mean *r *= 0.568, 95% CI: 0.326–0.721; *H*_o_: mean *r* = 0.431, 95% CI: 0.173–0.562).

### 3.2 Genetic differentiation among populations

Pairwise *F*_ST_ calculations among Brook Trout sampling localities revealed high levels of genetic differentiation, with an average *F*_ST_ across all pairwise comparisons of 0.238. Overall, sites within the Genesee basin displayed the highest levels of differentiation (mean *F*_ST_ = 0.206), followed by the Erie/Niagara (mean *F*_ST_ = 0.205), the Allegheny (mean *F*_ST_ = 0.196), and the Susquehanna (mean *F*_ST_ = 0.164). Differences among the four major basins in within‐basin *F*_ST_ values were statistically significant (*p* < 0.001). Pairwise comparisons between basins revealed the Susquehanna to be significantly different from the other basins (Genesee: *p* < 0.001; Allegheny: *p* = 0.003; Erie/Niagara: *p* = 0.046), while there was no statistical difference in pairwise comparisons between the Allegheny, Erie, and Genesee.

**Figure 3:**
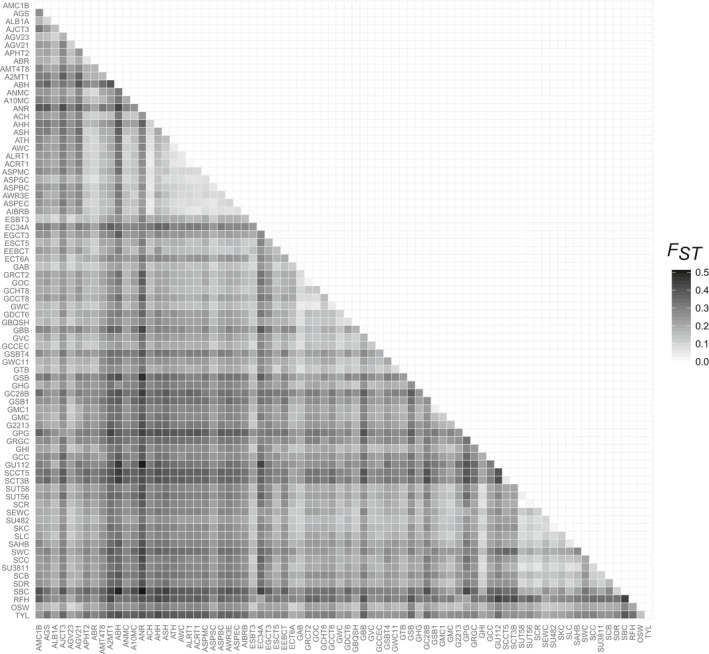
Pairwise *F*_ST_ matrix across all wild Brook Trout populations and hatchery strains. *F*_ST_ values are represented by a color gradient, with high values indicated by darker shades and low values indicated by lighter shades

All pairwise *F*_ST _values, except one, were statistically significant (Bonferroni‐corrected *p* = 0.00002), based on 10,000 simulations (results are available in Dryad). The only nonsignificant comparison was found for sites SUT58 and SCR, with an *F*_ST_ value of 0.00435 (*p* = 0.03). Nine wild populations exhibited overall high levels of differentiation, with mean *F*_ST_ values over 0.30 across all pairwise comparisons, including Allegheny populations ANR (mean *F*_ST_ = 0.349) and ABH (mean *F*_ST_ = 0.333), Erie/Niagara population EC34A (mean *F*_ST_ = 0.304), Genesee populations GPG (mean *F*_ST_ = 0.318) and GC28B (mean *F*_ST_ = 0.305), Susquehanna populations SBC (mean *F*_ST_ = 0.324), SCT3B (mean *F*_ST_ = 0.308), and SCCT5 (mean *F*_ST_ = 0.307), and Lake Ontario population GU112 (mean *F*_ST_ = 0.303). The Rome strain (ROM) also showed high levels of differentiation when compared to the wild populations (mean *F*_ST_ = 0.323). These patterns can be visualized in Figure 3 where *F*_ST_ values are represented by a gradient from dark shades (high differentiation) to light shades (low differentiation). Populations with overall high levels of differentiation exhibited dark shades across a majority of their associated pairwise comparisons.

Pockets of low differentiation can also be visualized in the *F*_ST_ heat map by the clusters of light gray shades along the diagonal. These within‐watershed comparisons are associated with populations within the Middle and Lower Allegheny River watersheds (mean *F*_ST_ = 0.127), sites in the Cryder Creek and Dyke Creek watersheds within the Genesee basin (mean *F*_ST_ = 0.113), and sites in the Upper Cohocton watershed within the Susquehanna basin (mean *F*_ST_ = 0.142), indicating a higher degree of connectivity within these watersheds.

**Figure 4:**
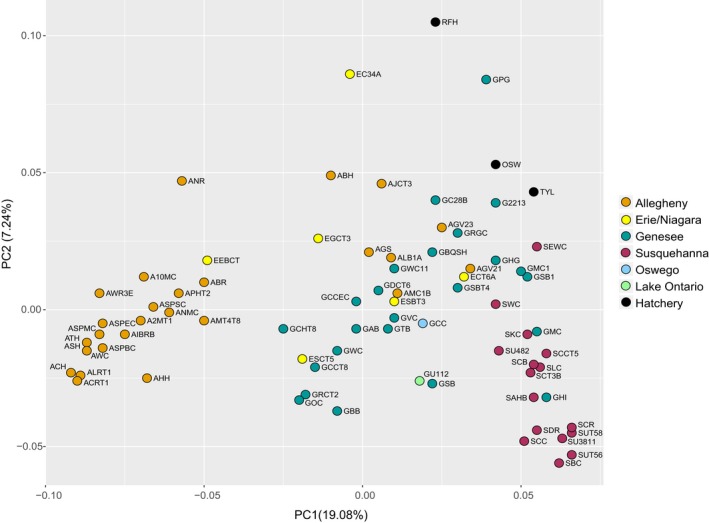
Principal coordinate analysis (PCoA) based on pairwise *F*_ST_ values between Brook Trout populations in western New York State. Points are labeled with their assigned population ID, and colors denote separate drainage basins or hatchery strains

The patterns across *F*_ST_ values were further visualized with the PCoA (Figure 4). The first two principal coordinates explained 26.32% of the variation. In general, localities within the same major drainage basin formed tighter clusters than sites located in separate basins. However, within a given basin, geographically isolated localities tended to be more genetically distant, diminishing the within‐basin genetic signatures, while geographically close populations formed tighter clusters. This concept can be observed within the Allegheny basin, where localities AJCT3, AGS, ALB1A, AMC1B, AGV23, and AGV21 were the most geographically distant sites and were distributed farther apart in the PCoA plot than the remainder of the Allegheny basin sites. One exception was site ANR, which is located in close proximity to many other Brook Trout populations, yet shows high levels of genetic partitioning.

The AMOVA showed that as the scale of the hydrologic unit decreased, combining the populations into smaller and smaller hydrologic regions, the percentage of variation explained by the groupings increased (Table 4). For all hydrologic scales except the HUC 12 level, the percentage of variation among groups was lower than the percentage of variation among populations within groups, likely due to the low level of gene flow at the population level. Because many of the HUC 12 subwatersheds contained only a single Brook Trout population, the higher percentage of among‐group variation at this hydrologic scale was likely due to the contribution of variation at the population level.

**Table 4:** Analysis of molecular variance (AMOVA) results among wild Brook Trout populations in western New York State. Populations were grouped into drainages at 6‐digit, 8‐digit, 10‐digit, and 12‐digit hydrologic unit codes (HUC) to assess hierarchical patterns of genetic variance

Source of variation	Sum of squares	Variance components	Percentage of variation	Statistics	*p*‐value
Grouped by basin (HUC 6)					
Among groups	2,251.982	0.379	7.511	*F_CT_* = 0.075	<0.0001
Among populations within groups	5,828.815	0.885	17.529	*F*_SC_ = 0.190	<0.0001
Within populations	25,491.701	3.786	74.960	*F*_ST_ = 0.250	<0.0001
Grouped by subbasin (HUC 8)					
Among groups	2,866.046	0.415	8.233	*F_CT_* = 0.082	<0.0001
Among populations within groups	5,214.751	0.84365	16.723	*F*_SC_ = 0.182	<0.0001
Within populations	25,491.701	3.78593	75.044	*F*_ST_ = 0.250	<0.0001
Grouped by watershed (HUC 10)					
Among groups	4,705.638	0.464	9.320	*F_CT_* = 0.093	<0.0001
Among populations within groups	3,375.160	0.728	14.628	*F*_SC_ = 0.161	<0.0001
Within populations	25,491.701	3.786	76.052	*F*_ST_ = 0.239	<0.0001
Grouped by subwatershed (HUC 12)					
Among groups	6,786.900	0.621	12.522	*F_CT_* = 0.125	<0.0001
Among populations within groups	1,293.898	0.551	11.112	*F*_SC_ = 0.127	<0.0001
Within populations	25,491.701	3.786	76.366	*F*_ST_ = 0.236	<0.0001

Results from the individual‐based assignment test showed that individuals could be genetically assigned to the correct source population with a high degree of accuracy, where correctly assigned individuals are considered those assigned to the population where they were originally collected. Overall, 93.4% of Brook Trout individuals were correctly assigned to the population from which they were sampled (Table 5). The assignment test correctly identified 100.0% of the individuals in 33 out of the 78 Brook Trout populations examined, including hatchery strains, indicating a high degree of genetic differentiation for these populations. Consistent with the *F*_ST_ results, populations SUT58 and SCR were found to have the lowest percentages of correct assignments (58.0% and 65.3%, respectively). A majority of the incorrectly assigned individuals sampled from SUT58 were assigned to SCR and vice versa. Site SUT56 also displayed a low degree of correct assignments (69.6%), with incorrectly assigned individuals equally distributed between SUT58 and SCR. This low degree of resolution suggests that gene flow could be occurring at higher frequencies within these tributaries. None of the wild Brook Trout individuals were assigned to a hatchery strain and vice versa.

**Table 5:** Assignment test results for Brook Trout in western New York State. The number and percentage of correctly assigned individuals (those assigned to their population of origin) are shown, as well as the number of incorrectly assigned individuals with the population to which each was assigned in parentheses

Pop ID	Correct assignments		Incorrect assignments
	*N*	%	
AMC1B	7	100.0	0
AGS	8	100.0	0
ALB1A	48	100.0	0
AJCT3	50	100.0	0
AGV23	48	100.0	0
AGV21	12	92.3	1 (AGV23 = 1)
APHT2	44	89.8	5 (ABR = 4, ASPSC = 1)
ABR	48	96.0	2 (APHT2 = 1, ACH = 1)
AMT4T8	50	100.0	0
A2MT1	50	100.0	0
ABH	30	100.0	0
ANMC	50	100.0	0
A10MC	50	100.0	0
ANR	50	100.0	0
ACH	39	78.0	11 (ALRT1 = 4, AWC = 2, ASPEC = 1, AHH = 2, ACRT1 = 1, AIBRB = 1)
AHH	48	98.0	1 (AIBRB = 1)
ASH	49	100.0	0
ATH	45	90.0	5 (AWC = 4, ABR = 1)
AWC	46	92.0	4 (ACH = 2, ACRT1 = 1, AIBRB = 1)
ALRT1	36	73.5	13 (ACRT1 = 7, AIBRB = 2, AWC = 2, ACH = 1, ANR = 1)
ACRT1	41	87.2	6 (ALRT1 = 5, AIBRB = 1)
ASPMC	46	92.0	4 (ASPEC = 2, ASPSC = 1, ANMC = 1)
ASPSC	49	98.0	1 (ASPEC = 1)
ASPBC	50	100.0	0
AWR3E	52	94.5	3 (ATH = 1, ACRT1 = 1, AWC = 1)
ASPEC	48	96.0	2 (ACH = 1, ASPMC = 1)
AIBRB	46	92.0	4 (AWC = 2, ABR = 1, ACH = 1)
ESBT3	49	98.0	1 (GBQSH = 1)
EC34A	49	98.0	1 (ABH = 1)
EGCT3	48	96.0	2 (GTB = 1, ALB1A = 1)
ESCT5	45	93.8	3 (GBQSH = 1, GWC = 1, GTB = 1)
EEBCT	51	100.0	0
ECT6A	50	100.0	0
GAB	50	98.0	1 (ALB1A = 1)
GRCT2	39	78.0	11 (GOC = 11)
GOC	42	84.0	8 (GRCT2 = 6, GAB = 1, GWC = 1)
GCHT8	30	90.9	3 (GAB = 2, GBQSH = 1)
GCCT8	47	95.9	2 (GWC = 2)
GWC	44	91.7	4 (GCCT8 = 2, GAB = 1, GRCT2 = 1)
GDCT6	13	86.7	2 (GBQSH = 2)
GBQSH	47	94.0	3 (GDCT6 = 1, GVC = 1, SCR = 1)
GBB	49	98.0	1 (GWC = 1)
GVC	47	97.9	1 (GBQSH = 1)
GCCEC	46	92.0	4 (ESBT3 = 2, GAB = 1, GHG = 1)
GSBT4	50	100.0	0
GWC11	50	100.0	0
GTB	49	98.0	1 (GAB = 1)
GSB	40	100.0	0
GHG	50	100.0	0
GC28B	50	100.0	0
GSB1	33	66.0	17 (GMC1 = 17)
GMC1	46	92.0	4 (GSB1 = 4)
GMC.	50	100.0	0
G2213	40	100.0	0
GPG	50	100.0	0
GRGC	48	100.0	0
GHI	30	93.8	2 (GBQSH = 1, SCC = 1)
GCC	49	100.0	0
GU112	20	100.0	0
SCCT5	44	88.0	6 (SCT3B = 6)
SCT3B	32	88.9	4 (SCCT5 = 4)
SUT58	29	58.0	21 (SCR = 15, SUT56 = 2, GHI = 2, SU3811 = 1, SU482 = 1)
SUT56	32	69.6	14 (SUT58 = 7, SCR = 7)
SCR	32	65.3	17 (SUT58 = 12, SUT56 = 4, SU3811 = 1)
SEWC	49	98.0	1 (SU482 = 1)
SU482	34	72.3	13 (SKC = 12, SCR = 1)
SKC	30	71.4	12 (SU482 = 12)
SLC	39	84.8	7 (SAHB = 6, SU482 = 1)
SAHB	47	94.0	3 (SLC = 2, SU3811 = 1)
SWC	49	100.0	0
SCC	49	100.0	0
SU3811	49	100.0	0
SCB	48	98.0	1 (SU3811 = 1)
SDR	48	96.0	2 (SU3811 = 1, GMC = 1)
SBC	31	100.0	0
ROM	50	100.0	0
OSW	50	100.0	0
TYL	50	100.0	0

**Figure 5:**
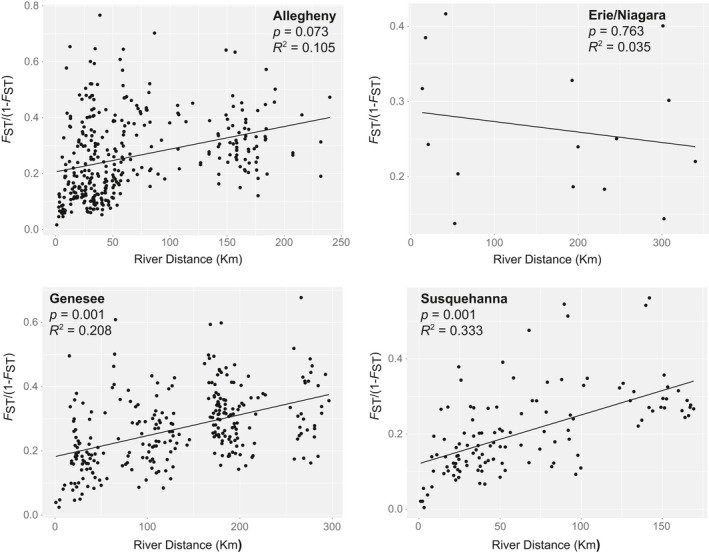
Isolation‐by‐distance (IBD) analysis showing the relationship between river distance and the linearized *F*_ST_ metric for each of the major drainage basins

The IBD analysis revealed a statistically significant linear relationship between river distance and genetic distance for the Genesee (*p* = 0.001, *R* ^2^ = 0.208) and the Susquehanna basins (*p* = 0.001, *R* ^2^ = 0.333). This relationship was approaching significance for the Allegheny basin (*p* = 0.073, *R* ^2^ = 0.105), but was not significant for the Erie/Niagara basin (Figure 5).

### 3.3 Population admixture

**Figure 6:**
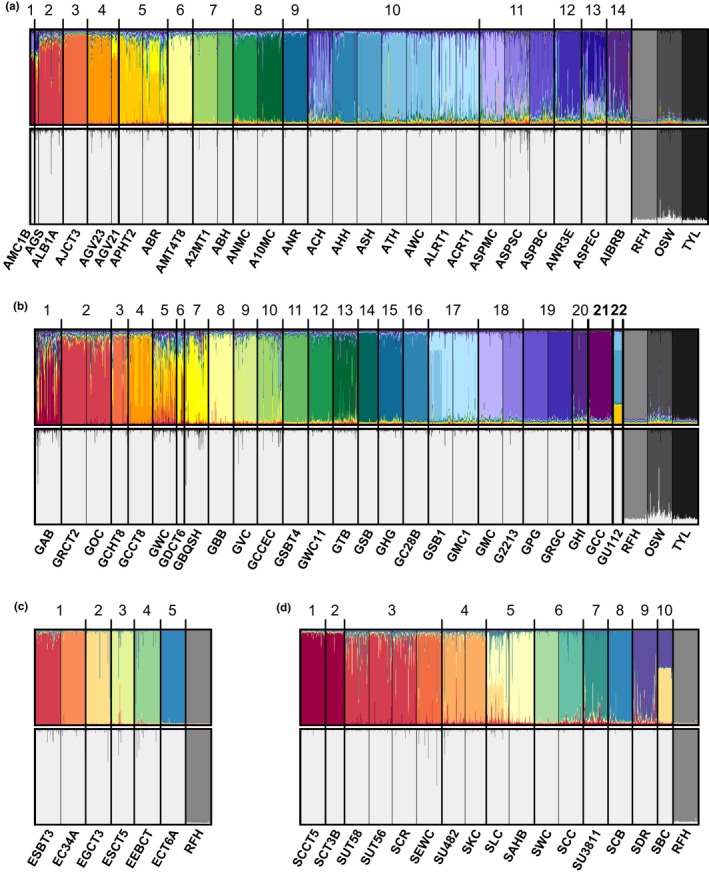
Bayesian clustering analysis of Brook Trout performed using the alternative parameter set, showing separate sampling localities labeled with their associated population ID, and subwatershed (12‐digit HUC level) delineations numbered corresponding to Table 1. For each basin, the full‐color plot is shown (top) as well as a plot with the wild Brook Trout genetic clusters all depicted in light gray (bottom) to better visualize hatchery contributions. (a) Allegheny basin: *K* = 25, (b) Genesee basin: *K* = 27, (c) Erie/Niagara basin: *K* = 7, and (d) Susquehanna basin: *K* = 12

A Bayesian clustering analysis was used to examine localities within each major drainage basin, along with the appropriate hatchery strains, to assess the degree of genetic admixture among populations as well as to infer the level of hatchery introgression. The default parameter set, using the uncorrelated allele frequency model and a uniform prior distribution for alpha, resulted in poor resolution between the Rome strain and some wild populations in the Allegheny (AMC1B), Erie/Niagara (population EC34A), and Genesee (population GPG) basins, making inferences of hatchery introgression inconclusive (Figures A3 and A4 in Appendix S2). Because these populations have no history of stocking (EC34A) or have not been directly stocked in over 60 years (AMC1B and GPG), this clustering pattern is not likely due to wild‐hatchery introgression. STRUCTURE was also unable to differentiate the Oswayo and Tylersville strains for the Allegheny basin analysis when the default parameters were applied (Figure A4 in Appendix S2). However, the alternative parameter set recommended by Wang (2017) drastically improved the clustering resolution, with all hatchery strains assigned to separate genetic clusters, and no wild populations completely assigned to a hatchery strain (Figure 6). Therefore, the results discussed hereafter will be based on the alternative Wang (2017) parameters.

For the Allegheny basin data set, which consisted of 27 sites and three hatchery strains, we concluded that the optimal number of genetic clusters, *K*, was 25, which was the *K*_MAX_ value, where *K*_MAX_ + 1 did not produce additional population groupings (Figure A5 in Appendix S2). A majority of the sampling locations were assigned to separate genetic clusters, with the exception of AGS/ALB1A, AGV23/AGV21, APHT2/ABR, ATH/AWC, and ALRT1/ACRT1 (Figure 6a), which are geographically proximate sites located within the same subwatershed. Consistent with the genetic diversity and *F*_ST_ results, a higher degree of wild‐wild admixture was observed in the Middle and Lower Allegheny River watersheds, which are comprised of the subwatersheds Windfall Creek, Red House Brook, Wolf Run, and Quaker Run (labeled 10–13). Population ACH, which exhibited the highest level of genetic diversity in the Allegheny basin, showed a high degree of admixture, even with nonadjacent sites. Similarly, other high diversity populations were found to show elevated levels of admixture, such as ALRT1, while low diversity populations showed little admixture (ABH, ANR, and AJCT3).

For the Erie/Niagara basin, we concluded that the six localities, in addition to the Rome strain, formed seven genetic clusters (Figure 6c, Figure A5 in Appendix S2). All populations were assigned to distinct clusters, showing little admixture. For both EC34A and ECT6A, which exhibited the lowest diversity levels for the Erie/Niagara basin, individuals were almost exclusively assigned to their respective genetic clusters, with little indication of interpopulation gene flow.

The Genesee basin sample localities (*N* = 24) were analyzed together with localities in the Oswego (GCC) and Lake Ontario (GU112) basins, in addition to the three hatchery strains, and produced 27 genetic clusters (Figure 6b, Figure A5 in Appendix S2). Geographically proximate populations, including GRCT2/GOC, GDCT6/GBQSH, and some individuals of GCCT8/GWC and GSB1/GMC1, were assigned to the same genetic clusters, while the remaining localities formed distinct clusters. A substantial degree of admixture was observed for populations within the Cryder Creek and Dyke Creek watersheds (comprised of subwatersheds labeled 2–7), and for localities GAB and GWC in particular, similar to the genetic diversity and *F*_ST_ results. In contrast, populations GSBT4 and GSB, which displayed low diversity levels, were also found to have low levels of admixture. Population GU112, located in the Lake Ontario basin, could not be consistently assigned to a single genetic cluster, but rather showed proportions of membership to three different clusters.

The 16 Susquehanna basin localities, combined with the Rome strain, formed 12 genetic clusters (Figure 6d, Figure A5 in Appendix S2). Localities within the Canacadea Creek watershed, SCCT5 and SCT3B, formed a single genetic cluster and displayed little admixture with the remaining sites. Localities SUT58, SUT56, and SCR found within the Punky Hollow subwatershed (labeled 3) grouped together in the same genetic cluster, reflecting the low *F*_ST_ values and high rates of incorrect assignment observed at these localities. Populations located in the Reynolds Creek subwatershed (labeled 4), sites SU482 and SKC, were also assigned to a single genetic cluster, and partial membership to the same cluster was observed for localities SLC/SAHB (Twelvemile Creek subwatershed, labeled 5) and SDR/SBC (Dry Run and Cutler Creek subwatersheds, labeled 9 and 10, respectively).

### 3.4 Hatchery introgression

Across all analysis methods, we found hatchery introgression, defined as having membership proportions (*q*‐values) above 0.10 to a genetic cluster associated with a hatchery strain, to be minimal. The basin‐wise STRUCTURE analyses using the alternative parameter set detected hatchery introgression in 1.96% of all wild Brook Trout individuals. We found similar results for the population‐specific analyses, examining each population individually along with the appropriate hatchery strain(s), which identified hatchery introgression in 3.10% and 2.08% of wild Brook Trout, applying the default and alternative parameter sets, respectively. Across all methods, a majority of the hatchery assignments were associated with the Rome strain (1.29%–1.73%) followed by the Oswayo strain (0.47%–1.20%) and the Tylersville strain (0.15%–0.18%; Tables A2–A4 in Appendix S1; Figure A6 in Appendix S2). By drainage basin, wild‐hatchery introgression was consistently detected at the lowest frequencies in the Susquehanna basin (3–7 individuals), followed by the Erie/Niagara basin (7–10 individuals), while the Allegheny (26–46 individuals) and Genesee (24–44 individuals) were found to have the highest hatchery introgression levels (Figure A6 in Appendix S2). We did not find evidence of wild‐hatchery introgression in either of the sites within the Oswego or Lake Ontario drainage basins.

Population ASPSC, located in Allegany State Park within the Allegheny basin, was found to have one of the highest mean assignment proportions to a hatchery strain, the Rome strain (mean *q* = 0.029–0.045), with multiple individuals showing evidence of wild‐hatchery introgression (Tables A2–A4 in Appendix S1). Membership proportions to the Oswayo and Tylersville hatchery strains were consistently detected in populations ALB1A, GBQSH, and GAB (Tables A2–A4 in Appendix S1).

Our linear regression analysis revealed a negative relationship between hatchery introgression in the wild Brook Trout populations examined (mean *q*‐values associated with the Rome hatchery strain) and the number of years since stocking occurred by the NYDEC. Although this trend was not statistically significant for any of the methods (basin‐wide STRUCTURE analysis with the alternative parameters and population‐specific STRUCTURE analyses using the default and alternative parameters), the mean hatchery *q*‐values calculated with the basin‐wide analysis showed a negative relationship that was approaching significance (*p* = 0.059, *R* ^2^ = 0.068).

From our analysis of simulated wild‐hatchery introgression, we found that after only four generations of backcrossing with a wild population (with equal *N*_e_ values of 50), membership proportions associated with the hatchery strain did not exceed 0.10 for any of the offspring, suggesting that hatchery introgression was below detectable levels (Figure A7 in Appendix S2). After three generations of backcrossing, only 4% of the offspring showed evidence of hatchery introgression.

## 4 DISCUSSION

With the increased fragmentation of Brook Trout populations due to habitat alterations (Timm et al., 2016; Torterotot et al., 2014; Whiteley et al., 2013), extirpation of nearby populations (Letcher et al., 2007), and thermal barriers associated with increased temperatures (Aunins et al., 2015), population monitoring is essential to minimize the rate of species decline. Incorporating genetic tools into population assessments can reveal previously unrecognized barriers to dispersal and identify population‐level relationships that can help inform management delineations and translocation decisions. Beyond population size estimates, metrics such as genetic diversity and effective population size, indicative of long‐term evolutionary potential, can aid in prioritizing preservation and remediation efforts. Here, we provide a detailed assessment of neutral genetic diversity for Brook Trout populations across a broad geographic range in New York State that can be utilized in conservation management and act as a baseline for continued population monitoring.

### 4.1 Genetic diversity, differentiation, and admixture

Previous studies examining Brook Trout populations in predominantly high‐quality habitats with little anthropogenic disturbances have found varying levels of genetic diversity. In the Adirondack watershed in northern New York State, Bruce, Hare, Mitchell, and Wright (2017) found a mean *A*_R_ of 5.35 (3.82–6.35, minimum sample size of 31) and a mean *H*_e_ of 0.598 (0.454–0.649). Brook Trout in minimally disturbed areas of New Hampshire were found to have a mean *A*_R_ of 5.4 (3.3–6.8, minimum sample size of 20) and a mean *H*_e_ of 0.56 (0.34–0.70) (Kelson et al., 2015). In contrast, Davis, Wagner, and Bartron (2015) found drastically higher diversity values for Brook Trout in north‐central Pennsylvania, exhibiting a mean *A*_R_ of 8.19 (7.81–8.50, minimum sample size of 36) and a mean *H*_e_ of 0.749 (0.736–0.758). Differences in the genetic diversity among relatively intact Brook Trout populations may reflect the heterogeneous nature of the headwater streams in which they reside, with some streams providing larger amounts of habitat than others, and therefore able to support larger Brook Trout populations.

Compared to the Brook Trout populations in high‐quality habitats described above, the populations in western New York State displayed slightly lower levels of allelic richness, but exhibited heterozygosity levels that were within the range of values reported in these studies. Although not statistically significant, the Allegheny basin was found to have the lowest mean diversity and effective population size estimates compared to the other major basins. One possible explanation for this observation is that the Allegheny contains a large number of sites that are geographically isolated from other Brook Trout populations due to extirpation. Across all basins, the relationship between population density and *A* was positive and approaching significance. Brook Trout populations with fewer neighboring populations, and thus fewer sources for new alleles, generally showed lower diversity values than those located in close proximity to many Brook Trout populations.

One example of a geographically isolated population is ABH, Bucher Hollow, which showed extremely low levels of allelic diversity and heterozygosity, represented in the box plots as an outlier or at the extreme end of the range (Figure A2 in Appendix S2). This site is located in the upstream portion of the Upper Allegheny River watershed and is secluded from a majority of the other populations examined. Additionally, only four adult Brook Trout and a large number of young‐of‐the‐year were observed at this site, explaining the high level of relatedness observed. Other examples include populations AJCT3 (Johnson Creek, T‐3) and A2MT1 (Twomile Creek), which are both geographically isolated and were found to have low levels of genetic diversity and high levels of genetic differentiation, evidenced by high *F*_ST_ values, 100% correct assignment rates, and limited admixture. Without the presence of neighboring populations due to local extirpation, gene flow is limited for many Brook Trout populations in the Allegheny basin and is likely contributing to the low diversity levels observed.

Populations can be isolated by factors other than distance, and barriers to fish passage play a large role in population fragmentation (Nathan, Kanno, & Vokoun, 2017; Timm et al., 2016; Torterotot et al., 2014). This is evident in population ANR, Newton Run, which exhibited low levels of diversity and was also represented in the box plots as an outlier or at the extreme low end of the range (Figure A2 in Appendix S2). This population displayed high levels of genetic isolation, yet is located in close proximity to a multitude of other Brook Trout populations. Several partial barriers were noted at this site during sample collection, as well as a potential complete barrier located at the mouth of the creek, which are likely impeding Brook Trout movement into and out of the population. Other studies have found Brook Trout genetic diversity to be negatively affected by barriers to fish movement, such as culverts and dams. Nathan et al. (2018) found that in Connecticut, Brook Trout populations located upstream of road culverts displayed lower diversity values (*A*_R_: 2.74–4.85, *H*_e_: 0.464–0.775, based on 17 populations) than populations unaffected by culverts (*A*_R_: 3.10–4.62, *H*_e_: 0.625–0.761, based on 11 populations). Torterotot et al. (2014) also described reduced genetic diversity in Brook Trout populations located upstream of barriers. In general, our study found that Brook Trout populations with a high degree of connectivity and wild–wild admixture tended to also exhibit higher levels of genetic variation than populations where little to no admixture was detected.

The Susquehanna basin displayed the highest overall levels of genetic diversity relative to the other three basins, and was found to have significantly lower within‐basin *F*_ST_ values, suggesting higher overall connectivity among sites. We found a significant positive relationship between population density, defined as the number of populations sampled within the same HUC 12 subwatershed, and all genetic diversity estimates for the Susquehanna sites. Aside from the two isolated populations in the Canacadea Creek watershed (SCCT5—Canacadea Creek, T‐5 and SCT3B—Canacadea Creek, T‐3B), the Susquehanna populations consistently showed moderate‐to‐high levels of allelic richness and heterozygosity, as well as large effective population sizes. These sites, predominantly located in the Upper Cohocton River watershed, were found to have low levels of genetic differentiation and high levels of incorrect assignment, suggesting high connectivity among populations. The only nonsignificant pairwise *F*_ST_ value detected in the data set was associated with a comparison between two sites in Punky Hollow subwatershed, SUT58 (an unnamed tributary) and SCR (Cohocton River), indicating that genetic partitioning was not detected between these two sites. Although Brook Trout have been extirpated from a large swath of the Susquehanna basin, the region that remains populated appears to have relatively high connectivity, which is likely contributing to the high levels of genetic diversity observed at these sites.

At a smaller scale, high connectivity was also detected within the Middle and Lower Allegheny watersheds within the Allegheny basin, as well as within the Cryder Creek and Dyke Creek watersheds within the Genesee basin, inferred from low *F*_ST_ values and high Bayesian admixture proportions. Sites within these watersheds also exhibited higher levels of genetic diversity. Gene flow among populations can maintain or increase the level of genetic variation within the populations involved, as new alleles are added and new genotypic combinations are created. This association between genetic diversity and population‐level admixture has been documented in other salmonids (Gomez‐Uchida, Knight, & Ruzzante, 2009; Van Leeuwen, Dalen, Museth, Junge, & Vøllestad, 2018; Matthaeus, 2016; Wofford, Gresswell, & Banks, 2005) as well as in Brook Trout populations in other regions (Bruce & Wright, 2018; Kanno, Vokoun, & Letcher, 2011; Kelson et al., 2015).

The linkage disequilibrium method of calculating *N*_e_ assumes a single, closed population (Waples & Do, 2008), an assumption which is violated when population admixture occurs. A genetic sample comprised of more than one gene pool results in a downward bias of *N*_e_, whereas scenarios in which migration increases the number of parents contributing to the local population cause an upward bias in *N*_e_ (Waples & England, 2011). For both of these scenarios, Waples and England (2011) concluded that the linkage disequilibrium method of calculating *N*_e_ is largely unaffected by migration. They found that the effects of additional parents to a local population, the most likely scenario for the New York Brook Trout populations, only skewed the *N*_e_ calculation when migration rates exceeded 5%–10% (Waples & England, 2011). Additionally, Waples and England (2011) determined that removing rare alleles from the calculation, a criterion applied in our analysis, effectively removed the bias associated with recent immigrants. Considering the relatively low degree of admixture and high degree of genetic differentiation that we observed for a majority of the Brook Trout populations examined, our *N*_e _calculations were not likely skewed by high rates of migration. Comparisons to other Brook Trout studies lend additional support to the low *N*_e_ estimates observed for western New York populations. Estimates of *N*_e_ for Brook Trout in northern New York ranged from 24.3 (14.0–50.6) to 296.7 (118–∞; Bruce et al., 2017), and populations in north‐central Pennsylvania exhibited *N*_e_ values ranging from 27.4 (23.7–32.5) to 99.4 (60.4–234.4; Davis et al., 2015).

Our study found the Susquehanna and Genesee basins to exhibit a statistically significant effect of IBD, the positive linear relationship between geographic distance and genetic differentiation due to limited dispersal. This result suggests that river distance is strongly contributing to the degree of gene flow among Brook Trout populations in these drainage basins. We found a positive, but not statistically significant, IBD trend for Brook Trout in the Allegheny basin; however, populations in the Erie/Niagara did not show a positive relationship between river distance and genetic distance. Departure from IBD can occur when barriers to migration disrupt the dispersal abilities of a population, leading to higher than expected levels of genetic differentiation (Frantz, Pope, Etherington, Wilson, & Burke, 2010; Meirmans, 2012). The tributaries of the Erie/Niagara basin feed into Lake Erie, which is likely acting as a dispersal barrier for stream‐dwelling Brook Trout, and eroding the IBD pattern. Similarly, many Brook Trout populations within the Allegheny basin are separated by barriers in the form of extirpated regions, which may also decrease the strength of the IBD signal.

### 4.2 Hatchery introgression

The software program STRUCTURE is commonly used to detect population subdivisions within a data set, but has been shown to produce erroneous population assignments when unbalanced sample sizes occur and when *K* values are very large (Kalinowski, 2011; Wang, 2017). A simulation study by Kalinowski (2011) also showed that when population divergence times were relatively long, the clustering patterns produced by STRUCTURE were often inconsistent with the true evolutionary history. The presence of hatchery strains in our analysis, which are presumably more distantly related and thus have longer divergence times, in addition to the large *K* values examined, likely prompted the incorrect clustering patterns observed under the default parameters. The alternative parameter set suggested by Wang (2017) greatly improved the clustering patterns, showing greater resolution among the three hatchery strains as well as among wild and hatchery individuals. Additionally, analyzing each population independently with the appropriate hatchery strain(s) corroborated the results from the basin‐wide analyses using the alternative parameters. This provided increased confidence that the clustering patterns produced with the alternative Wang (2017) parameters were a more accurate representation of the true biological processes occurring. Our study highlights the need to carefully select the appropriate parameters for a given analysis and not rely solely on the default settings to provide accurate results.

Despite concerns of historic widespread Brook Trout stocking in New York State and the potential for genetic swamping of stocked populations (Perkins et al., 1993), we found evidence for limited hatchery introgression. Only 1.96%–3.10% of the Brook Trout individuals examined showed signs of hatchery introgression. This result is consistent with other studies on wild, stream‐dwelling Brook Trout populations, which have found hatchery introgression levels to be minimal (Annett, Gerlach, King, & Whiteley, 2012; Kelson et al., 2015; White et al., 2018). Variables such as stocking intensity (Marie et al., 2010), availability of high‐quality habitat (Marie et al., 2012), and the amount of time since stocking occurred (Létourneau et al., 2017) have been shown to be correlated with the degree of hatchery introgression in Brook Trout. In natural environments, hatchery fish also tend to exhibit lower survival rates (Baer, Blasel, & Diekmann, 2007; Fraser, 1981) and lower reproductive abilities (Araki, Berejikian, Ford, & Blouin, 2008; Araki, Cooper, & Blouin, 2007), which could also explain the low level of hatchery introgression observed.

Recent stocking of the Rome Brook Trout strain has occurred in multiple tributaries within Allegany State Park, including Red House Brook (1986–2008: annual mean = 2,215), which is the receiving stream for Stoddard Creek (ASPSC), Beehunter Creek (ASPBC), and McIntosh Creek (ASPMC), which could explain the higher rate of introgression observed in these populations. Similarly, high proportions of membership to Pennsylvania State Fish Hatchery strains, the Oswayo and Tylersville strains, were found in the Allegheny basin (populations ALB1A) and the Genesee basin (populations GBQSH and GAB). These sites are all in close proximity to the Pennsylvania border, and potential hatchery introgression could be explained by nearby stocking locations along with the migration or human‐mediated dispersal of Brook Trout into New York State.

Our study found a negative trend between the number of years since stocking occurred and the degree of wild‐hatchery introgression. Although approximately half of the populations examined in this study have been directly stocked in the past, most have not been stocked since the 1970s, and none of the sites are currently stocked. This suggests that if hatchery introgression was occurring in the past, the genetic signatures have been diluted to a low or nondetectable level after generations of backcrossing with wild individuals. Our simulation analysis confirmed this hypothesis, showing that after four generations of backcrossing with wild individuals, genetic signatures of hatchery introgression were no longer detected. This rapid dilution of hatchery alleles occurred when sample sizes were equal between hatchery/wild and hybrid/wild individuals, a scenario that would likely not occur in nature. Rather, sample sizes would likely be skewed toward fewer hatchery and hybrid individuals compared to wild individuals, further hastening the dilution effect of the hatchery alleles.

One caveat to our analysis is that the Rome Brook Trout strain stocked in our study streams decades ago may not be genetically equivalent to the Rome samples currently examined due to years of genetic drift. Because historic specimens of the Rome Brook Trout strain were not available, we could not directly compare their genotypic profile against modern samples. However, the negative trend between hatchery introgression and stocking time, as well as the results of our simulated wild‐hatchery introgression and backcrossing analysis, provides increased confidence that the pattern of hatchery strain dilution in wild populations over time, as inferred in our study, reflects true processes and is not simply an artifact of genetic drift skewing the genotypic profile of hatchery individuals.

## 5 CONCLUSIONS

Although Brook Trout populations often show high degrees of differentiation, even within interconnected stream networks, isolated populations are at risk of reduced genetic diversity and inbreeding. With the widespread extirpation of Brook Trout throughout western New York State (Eastern Brook Trout Joint Venture, 2016), remaining populations have become increasingly isolated, with fewer neighboring tributaries able to act as sources of new alleles. Our results suggest that watersheds containing higher densities of Brook Trout populations tended to display higher levels of admixture and genetic diversity than watersheds with few sites isolated by far distances. As described in the simulation study by Letcher et al. (2007), following local extirpation of Brook Trout populations, the likelihood of system‐wide extinctions increased due to the lack of neighboring tributaries that could function as population sources. Isolated populations can persist, however, provided the amount of available high‐quality habitat is sufficiently large (Whiteley et al., 2013). Therefore, action should be taken to improve the habitat of existing Brook Trout populations, as well as increase the amount of available habitat by eliminating man‐made barriers to fish movement, when appropriate.

One of the principal goals of the Eastern Brook Trout Joint Venture is to identify and protect functional, stable Brook Trout populations and the surrounding habitat (Eastern Brook Trout Joint Venture, 2011). Robust populations, identified by having both high genetic diversity and large effective population sizes, should be considered a high priority for protection, while populations showing signs of isolation should take precedence for habitat assessments and restoration. Although this study only examined neutral genetic variation, adaptive variation may also be important for conservation management. As a cold‐water species, the ability of Brook Trout populations to tolerate warming water temperatures will become increasingly important for population persistence in the face of climate change. Studies have found wide variation among Brook Trout populations in their ability to acclimate to warmer temperature regimes, and this response was associated with variation in the expression of heat shock proteins (Stitt et al., 2014). Therefore, future Brook Trout conservation strategies may also consider taking heat tolerance into account when setting management priorities.

Assessments of neutral genetic variation, such as this study, can be useful in identifying populations to focus conservation efforts, and future investigations can make comparisons to the baseline values presented here to track the progress of Brook Trout conservation. Brook Trout are considered to be a keystone species in many headwater streams, holding the highest trophic position, and causing trophic cascades that affect the abundance of a wide range of organisms from insectivorous fish and salamanders to detritivorous insects (Tzilkowski, 2005). In addition to their role in the food chain, Brook Trout are representative of high‐quality headwater streams, and therefore, preserving and restoring Brook Trout habitat will benefit not only the species, but also the ecosystem as a whole.

## CONFLICT OF INTEREST

None declared.

## AUTHOR CONTRIBUTIONS

The authors made the following contributions: Stephanie Dowell Beer: performed laboratory work, analyses, and wrote the manuscript; Scott Cornett: involved in project design and planning, sample collection, and manuscript review; Peter Austerman: involved in project design and planning, sample collection, and manuscript review; Betsy Trometer: involved in project design and planning, sample collection, manuscript review, and provided funding; Thomas Hoffman: involved in project design, planning, and sample collection; and Meredith Bartron: involved in project design and planning, coordination of funding, and manuscript review.

## Data Availability

Sampling location GPS coordinates, microsatellite genotypes, and *F*_ST_ results are available through the Dryad database (https://doi.org/10.5061/dryad.bp8dn88).
