# Supplementary material for: Genetic diversity, admixture, and hatchery influence in Brook Trout (Salvelinus fontinalis) throughout western New York State
Source: Ecol Evol. 2019 Jun 14;9(13):7455–79. doi: 10.1002/ece3.5237 (PMC6635958; doi:10.1002/ece3.5237)
Supplement: Supplementary file 1 [file ECE3-9-7455-s001.docx]

**Table A1** Stocking information showing the years that New York State Department of Environmental Conservation stocked Rome strain Brook Trout either directly into the study streams, or into the receiving stream, defined as an adjacent stream directly connected to the study stream. Stocking in Pennsylvania refers to the years that Pennsylvania Fish and Boat commission stocked Oswayo or Tylersville strain Brook Trout.

| **Stream** | **ID** | **Basin** | **Years Stocked** | **Years Receiving Stream Stocked** |
| --- | --- | --- | --- | --- |
| Mill Creek, T-1B | AMC1B | Allegheny | 1932,33,36 | 2010-2016 |
| Gallop (Town) Stream | AGS | Allegheny | 1928,37-92 | NA - dam above mouth |
| Little Brownell, T-1A | ALB1A | Allegheny | None | 1955-65, 1968-92 |
| Johnson Creek, T-3 | AJCT3 | Allegheny | None | None |
| Great Valley Creek, T-23 | AGV23 | Allegheny | 1930-35, 1973 | None |
| Great Valley Creek, T-21 | AGV21 | Allegheny | None | None |
| Pumplin Hollow & T-2 | APHT2 | Allegheny | None | 1931 |
| Barker Run | ABR | Allegheny | 1928-30,32 | 1931 |
| Mix Creek & T-1, T-8 | AMT4T8 | Allegheny | 1932 | None |
| Two Mile Creek & T-1 | A2MT1 | Allegheny | None | None |
| Bucher Hollow | ABH | Allegheny | None | None |
| Nine Mile Creek | ANMC | Allegheny | 1927-65 | None |
| Ten mile Creek | A10MC | Allegheny | 1928-36 | None |
| Newton Run | ANR | Allegheny | None | None |
| Christian Hollow | ACH | Allegheny | None | None |
| Hardscrabble Hollow | AHH | Allegheny | None | None |
| Sullivan Hollow | ASH | Allegheny | None | None |
| Thorpe Hollow | ATH | Allegheny | 1957 | None |
| Windfall Creek | AWC | Allegheny | 1927-1957 | None |
| Leonard Run and T-1 | ALRT1 | Allegheny | None | None |
| Carrollton Run and T-1 | ACRT1 | Allegheny | None | None |
| McIntosh Creek | ASPMC | Allegheny | 1935, 1949? | 1955-79,80,81,83-89,91-94,1996-2008 |
| Stoddard Creek | ASPSC | Allegheny | 1949 | 1955-79,80,81,83-89,91-94,1996-2008 |
| Beehunter Creek | ASPBC | Allegheny | 1955-77,79,80,83-86,88,89,91-2004 | 1955-79,80,81,83-89,91-94,1996-2008 |
| Wolf Run and T-3 | AWR3E | Allegheny | 1928-55,1959-67,69-74,76,77,80,81,83-86 | None |
| English Creek | ASPEC | Allegheny | 1949 | 1955-81,83-89,91-93,1996-2008 |
| Irish and Rice Brooks | AIBRB | Allegheny | 1928,37-65 (Rice), Irish-none | None |
| Spring Brook & T-3 | ESBT3 | Erie/Niagara | 1882, 1937-51, 55, 56, 62 | None |
| Cattaraugus Creek, T-34A | EC34A | Erie/Niagara | None | None |
| Gooseneck Creek & T-3 | EGCT3 | Erie/Niagara | 1966-68 | NA - falls above mouth |
| Spring Creek &T-5 | ESCT5 | Erie/Niagara | 1878, 1882 | None |
| E. Br. Cazenovia Ck trib | EEBCT | Erie/Niagara | None | 1927? |
| Crow Creek & T-6A | ECT6A | Erie/Niagara | 1882 | NA - dam above mouth |
| Ainsworth Brook | GAB | Genesee | None | None |
| Redwater Creek & T-2 | GRCT2 | Genesee | 1926-55 | 2014 in Pennsylvania |
| Orebed Creek | GOC | Genesee | 1881,1889,1926-55 | 2014 in Pennsylvania |
| Chenunda Creek, T-8 | GCHT8 | Genesee | 1926-41 | 1946 |
| Cryder Creek, T-8 | GCCT8 | Genesee | None | 1955,59-67,69-77,79-86 |
| Wileyville Creek | GWC | Genesee | 1942-70 | 1986 in R8 |
| Dyke Creek, T-6 | GDCT6 | Genesee | None | 1926? |
| Best, Quig & Shovel Hollow | GBQSH | Genesee | 1926, 41,71-73 (Shovel) | 1926? |
| Brimmer Brook | GBB | Genesee | 1878-79 | 2014 in Pennsylvania |
| Vandermark Creek | GVC | Genesee | 1953,75,83-89,92-94 | 2014 in Pennsylvania |
| Cold Creek and Elm Creek | GCCEC | Genesee | 1926, 1971 (Elm Ck) | None |
| Spencer Bk. T-4 of Wiscoy | GSBT4 | Genesee | None | NA - falls above mouth |
| Wiscoy Creek, T-11 | GWC11 | Genesee | None | 1926 |
| Trout Brook and tribs | GTB | Genesee | 1926-55 | 1926 |
| Spring Brook | GSB | Genesee | None | None |
| Hovey Gully | GHG | Genesee | 1941 -1953 | 1941 - 1953 |
| Canaseraga Creek, T-28B | GC28B | Genesee | None | None |
| Stony Brook | GSB1 | Genesee | 1956 - 1971 | 1971 |
| Mill Creek | GMC1 | Genesee | 1956 - 1971 | 1971 |
| Mill Creek | GMC | Genesee | 1941 -1948 | 1941 - 1953 |
| Unnamed Tributary | G2213 | Genesee | 1958 - 1965 | 1941 - 1965 |
| Pokamoonshine Gulf | GPG | Genesee | 1941 - 1953 | 1952-1953 |
| Reynolds Gully Creek | GRGC | Genesee | None | None |
| Honeoye Inlet | GHI | Genesee | 1941 -1950 | None |
| Grimes Creek | GCC | Oswego | 1941 - 48,58 -63 | None |
| Unnamed Tributary | GU112 | Ontario | None | None |
| Canacadea Creek, T-5 | SCCT5 | Susquehanna | None | 1935 |
| Canacadea Creek, T-3B | SCT3B | Susquehanna | 1930? | 1935 |
| Unnamed Tributary | SUT58 | Susquehanna | None | None |
| Unnamed Tributary | SUT56 | Susquehanna | None | 1942,44-50, 52-78 |
| Cohocton River | SCR | Susquehanna | 1942,44-50, 52-78 | None |
| East Wayland Creek | SEWC | Susquehanna | 1941 -1965 | 1942,44-50, 52-78 |
| Unnamed Tributary | SU482 | Susquehanna | None | None |
| Kirkwood Creek | SKC | Susquehanna | None | 1942,44-50, 52-78 |
| Lyon Creek | SLC | Susquehanna | 1957 - 1970 | None |
| Avery Hollow Brook | SAHB | Susquehanna | None | 1957 - 1970 |
| West Creek | SWC | Susquehanna | None | None |
| Cotton Creek | SCC | Susquehanna | 1942 | 1942,44-50, 52-78 |
| Unnamed Tributary | SU3811 | Susquehanna | None | 1942,44-50, 52-78 |
| Chamberlain Brook | SCB | Susquehanna | None | None |
| Dry Run | SDR | Susquehanna | None | 1964 |
| Borden Creek | SBC | Susquehanna | None | None |

**Table A2** Membership proportions (*q*-values) of wild Brook Trout to hatchery strains, produced with the basin-wide STRUCTURE analysis using the alternative parameter set. The mean, minimum, and maximum *q*-values to each hatchery cluster are reported for each population, along with the number of individuals displaying *q*-values > 0.1, indicating potential hatchery introgression.

| **ID** | **ROM** | | | | **OSW** | | | | **TYL** | | | |
| --- | --- | --- | --- | --- | --- | --- | --- | --- | --- | --- | --- | --- |
|  | **Mean *q*** | **Min *q*** | **Max *q*** | ***q* > 0.1** | **Mean *q*** | **Min *q*** | **Max *q*** | ***q* > 0.1** | **Mean *q*** | **Min *q*** | **Max *q*** | ***q* > 0.1** |
| AMC1B | 0.005 | 0.002 | 0.011 | 0 | 0.007 | 0.002 | 0.017 | 0 | 0.022 | 0.007 | 0.046 | 0 |
| AGS | 0.027 | 0.005 | 0.083 | 0 | 0.019 | 0.003 | 0.099 | 0 | 0.005 | 0.002 | 0.014 | 0 |
| ALB1A | 0.007 | 0.002 | 0.047 | 0 | 0.018 | 0.002 | 0.304 | 2 | 0.011 | 0.001 | 0.168 | 1 |
| AJCT3 | 0.004 | 0.002 | 0.026 | 0 | 0.003 | 0.001 | 0.005 | 0 | 0.002 | 0.001 | 0.006 | 0 |
| AGV23 | 0.028 | 0.002 | 0.404 | 3 | 0.014 | 0.002 | 0.143 | 1 | 0.004 | 0.002 | 0.018 | 0 |
| AGV21 | 0.012 | 0.003 | 0.042 | 0 | 0.011 | 0.004 | 0.057 | 0 | 0.009 | 0.003 | 0.031 | 0 |
| APHT2 | 0.008 | 0.002 | 0.032 | 0 | 0.004 | 0.001 | 0.025 | 0 | 0.003 | 0.001 | 0.014 | 0 |
| ABR | 0.015 | 0.002 | 0.194 | 2 | 0.006 | 0.002 | 0.060 | 0 | 0.004 | 0.002 | 0.016 | 0 |
| AMT4T8 | 0.005 | 0.002 | 0.060 | 0 | 0.004 | 0.001 | 0.015 | 0 | 0.005 | 0.001 | 0.030 | 0 |
| A2MT1 | 0.010 | 0.001 | 0.204 | 2 | 0.002 | 0.001 | 0.008 | 0 | 0.002 | 0.001 | 0.007 | 0 |
| ABH | 0.006 | 0.002 | 0.040 | 0 | 0.003 | 0.001 | 0.010 | 0 | 0.002 | 0.001 | 0.006 | 0 |
| ANMC | 0.007 | 0.002 | 0.094 | 0 | 0.004 | 0.001 | 0.013 | 0 | 0.004 | 0.001 | 0.013 | 0 |
| A10MC | 0.004 | 0.001 | 0.017 | 0 | 0.004 | 0.001 | 0.064 | 0 | 0.003 | 0.001 | 0.010 | 0 |
| ANR | 0.005 | 0.001 | 0.071 | 0 | 0.003 | 0.001 | 0.016 | 0 | 0.002 | 0.001 | 0.010 | 0 |
| ACH | 0.004 | 0.001 | 0.015 | 0 | 0.007 | 0.001 | 0.151 | 1 | 0.005 | 0.001 | 0.063 | 0 |
| AHH | 0.004 | 0.001 | 0.022 | 0 | 0.002 | 0.001 | 0.013 | 0 | 0.002 | 0.001 | 0.007 | 0 |
| ASH | 0.003 | 0.001 | 0.016 | 0 | 0.003 | 0.001 | 0.019 | 0 | 0.002 | 0.001 | 0.006 | 0 |
| ATH | 0.004 | 0.002 | 0.014 | 0 | 0.003 | 0.001 | 0.019 | 0 | 0.002 | 0.001 | 0.009 | 0 |
| AWC | 0.006 | 0.001 | 0.070 | 0 | 0.003 | 0.001 | 0.011 | 0 | 0.004 | 0.001 | 0.036 | 0 |
| ALRT1 | 0.004 | 0.001 | 0.031 | 0 | 0.005 | 0.001 | 0.050 | 0 | 0.004 | 0.001 | 0.031 | 0 |
| ACRT1 | 0.004 | 0.001 | 0.023 | 0 | 0.003 | 0.001 | 0.018 | 0 | 0.003 | 0.001 | 0.011 | 0 |
| ASPMC | 0.009 | 0.001 | 0.172 | 1 | 0.005 | 0.001 | 0.080 | 0 | 0.003 | 0.001 | 0.017 | 0 |
| ASPSC | 0.045 | 0.002 | 0.509 | 6 | 0.018 | 0.001 | 0.229 | 2 | 0.005 | 0.001 | 0.017 | 0 |
| ASPBC | 0.012 | 0.001 | 0.134 | 1 | 0.004 | 0.001 | 0.015 | 0 | 0.004 | 0.001 | 0.020 | 0 |
| AWR3E | 0.005 | 0.001 | 0.025 | 0 | 0.004 | 0.001 | 0.019 | 1 | 0.003 | 0.001 | 0.031 | 0 |
| ASPEC | 0.010 | 0.002 | 0.140 | 1 | 0.004 | 0.001 | 0.013 | 0 | 0.004 | 0.001 | 0.032 | 0 |
| AIBRB | 0.010 | 0.001 | 0.192 | 1 | 0.005 | 0.001 | 0.033 | 0 | 0.006 | 0.001 | 0.118 | 1 |
|  |  |  | **Total:** | **17** |  |  | **Total:** | **7** |  |  | **Total:** | **2** |
| GAB | 0.018 | 0.002 | 0.150 | 4 | 0.014 | 0.002 | 0.160 | 1 | 0.019 | 0.002 | 0.471 | 2 |
| GRCT2 | 0.003 | 0.001 | 0.013 | 0 | 0.003 | 0.001 | 0.011 | 0 | 0.004 | 0.001 | 0.019 | 0 |
| GOC | 0.003 | 0.001 | 0.016 | 0 | 0.009 | 0.001 | 0.116 | 2 | 0.005 | 0.001 | 0.046 | 0 |
| GCHT8 | 0.013 | 0.001 | 0.151 | 1 | 0.004 | 0.001 | 0.040 | 0 | 0.003 | 0.001 | 0.010 | 0 |
| GCCT8 | 0.003 | 0.001 | 0.023 | 0 | 0.003 | 0.001 | 0.016 | 0 | 0.003 | 0.001 | 0.018 | 0 |
| GWC | 0.009 | 0.002 | 0.041 | 0 | 0.008 | 0.002 | 0.058 | 0 | 0.013 | 0.002 | 0.132 | 1 |
| GDCT6 | 0.006 | 0.002 | 0.012 | 0 | 0.007 | 0.003 | 0.022 | 0 | 0.006 | 0.002 | 0.052 | 0 |
| GBQSH | 0.018 | 0.002 | 0.198 | 2 | 0.023 | 0.002 | 0.572 | 2 | 0.010 | 0.002 | 0.072 | 0 |
| GBB | 0.004 | 0.001 | 0.031 | 0 | 0.002 | 0.001 | 0.006 | 0 | 0.003 | 0.001 | 0.009 | 0 |
| GVC | 0.016 | 0.002 | 0.360 | 1 | 0.009 | 0.002 | 0.147 | 1 | 0.005 | 0.002 | 0.018 | 0 |
| GCCEC | 0.015 | 0.002 | 0.189 | 2 | 0.013 | 0.002 | 0.147 | 2 | 0.005 | 0.001 | 0.016 | 0 |
| GSBT4 | 0.004 | 0.002 | 0.012 | 0 | 0.004 | 0.002 | 0.021 | 0 | 0.004 | 0.002 | 0.011 | 0 |
| GWC11 | 0.006 | 0.001 | 0.041 | 0 | 0.006 | 0.002 | 0.041 | 0 | 0.004 | 0.001 | 0.027 | 0 |
| GTB | 0.008 | 0.002 | 0.058 | 0 | 0.006 | 0.002 | 0.042 | 0 | 0.005 | 0.001 | 0.024 | 0 |
| GSB | 0.002 | 0.001 | 0.003 | 0 | 0.002 | 0.001 | 0.009 | 0 | 0.003 | 0.001 | 0.013 | 0 |
| GHG | 0.007 | 0.001 | 0.083 | 0 | 0.006 | 0.002 | 0.035 | 0 | 0.006 | 0.002 | 0.048 | 0 |
| GC28B | 0.003 | 0.002 | 0.013 | 0 | 0.004 | 0.002 | 0.021 | 0 | 0.003 | 0.002 | 0.020 | 0 |
| GSB1 | 0.003 | 0.001 | 0.009 | 0 | 0.004 | 0.002 | 0.016 | 0 | 0.009 | 0.001 | 0.156 | 1 |
| GMC1 | 0.005 | 0.001 | 0.015 | 0 | 0.006 | 0.002 | 0.033 | 0 | 0.006 | 0.002 | 0.063 | 0 |
| GMC | 0.005 | 0.001 | 0.028 | 0 | 0.006 | 0.002 | 0.051 | 0 | 0.004 | 0.002 | 0.023 | 0 |
| G2213 | 0.006 | 0.002 | 0.026 | 0 | 0.007 | 0.002 | 0.115 | 1 | 0.004 | 0.002 | 0.039 | 0 |
| GPG | 0.006 | 0.002 | 0.037 | 0 | 0.003 | 0.002 | 0.007 | 0 | 0.003 | 0.002 | 0.014 | 0 |
| GRGC | 0.005 | 0.002 | 0.017 | 0 | 0.003 | 0.002 | 0.008 | 0 | 0.004 | 0.002 | 0.017 | 0 |
| GHI | 0.013 | 0.002 | 0.160 | 1 | 0.008 | 0.002 | 0.056 | 0 | 0.006 | 0.002 | 0.017 | 0 |
|  |  |  | **Total:** | **11** |  |  | **Total:** | **9** |  |  | **Total:** | **4** |
|  |  |  |  |  |  |  |  |  |  |  |  |  |
| GCC | 0.004 | 0.001 | 0.023 | 0 | 0.003 | 0.002 | 0.012 | 0 | 0.005 | 0.002 | 0.032 | 0 |
|  |  |  |  |  |  |  |  |  |  |  |  |  |
| GU112 | 0.002 | 0.001 | 0.003 | 0 | 0.002 | 0.001 | 0.006 | 0 | 0.003 | 0.002 | 0.008 | 0 |
|  |  |  |  |  |  |  |  |  |  |  |  |  |
|  |  |  |  |  |  |  |  |  |  |  |  |  |
| SCCT5 | 0.004 | 0.001 | 0.055 | 0 |  |  |  |  |  |  |  |  |
| SCT3B | 0.002 | 0.001 | 0.008 | 0 |  |  |  |  |  |  |  |  |
| SUT58 | 0.005 | 0.001 | 0.035 | 0 |  |  |  |  |  |  |  |  |
| SUT56 | 0.004 | 0.001 | 0.037 | 0 |  |  |  |  |  |  |  |  |
| SCR | 0.008 | 0.001 | 0.095 | 0 |  |  |  |  |  |  |  |  |
| SEWC | 0.031 | 0.002 | 0.593 | 4 |  |  |  |  |  |  |  |  |
| SU482 | 0.014 | 0.001 | 0.268 | 1 |  |  |  |  |  |  |  |  |
| SKC | 0.006 | 0.002 | 0.037 | 0 |  |  |  |  |  |  |  |  |
| SLC | 0.011 | 0.002 | 0.102 | 1 |  |  |  |  |  |  |  |  |
| SAHB | 0.004 | 0.001 | 0.014 | 0 |  |  |  |  |  |  |  |  |
| SWC | 0.004 | 0.001 | 0.020 | 0 |  |  |  |  |  |  |  |  |
| SCC | 0.004 | 0.001 | 0.031 | 0 |  |  |  |  |  |  |  |  |
| SU3811 | 0.004 | 0.001 | 0.030 | 0 |  |  |  |  |  |  |  |  |
| SCB | 0.007 | 0.001 | 0.104 | 1 |  |  |  |  |  |  |  |  |
| SDR | 0.004 | 0.001 | 0.014 | 0 |  |  |  |  |  |  |  |  |
| SBC | 0.003 | 0.001 | 0.013 | 0 |  |  |  |  |  |  |  |  |
|  |  |  | **Total:** | **7** |  |  |  |  |  |  |  |  |
|  |  |  |  |  |  |  |  |  |  |  |  |  |
| ESBT3 | 0.016 | 0.001 | 0.136 | 3 |  |  |  |  |  |  |  |  |
| EC34A | 0.013 | 0.002 | 0.247 | 1 |  |  |  |  |  |  |  |  |
| EGCT3 | 0.012 | 0.001 | 0.346 | 1 |  |  |  |  |  |  |  |  |
| ESCT5 | 0.012 | 0.001 | 0.272 | 2 |  |  |  |  |  |  |  |  |
| EEBCT | 0.017 | 0.001 | 0.290 | 3 |  |  |  |  |  |  |  |  |
| ECT6A | 0.003 | 0.001 | 0.007 | 0 |  |  |  |  |  |  |  |  |
|  |  |  | **Total:** | **10** |  |  |  |  |  |  |  |  |

**Table A3** Membership proportions (*q*-values) of wild Brook Trout to hatchery strains, produced with the population-specific STRUCTURE analysis using the alternative parameter set. The mean, minimum, and maximum *q*-values to each hatchery cluster are reported for each, along with the number of individuals displaying *q*-values > 0.1, indicating potential hatchery introgression.

| **ID** | **ROM** | | | | **OSW** | | | | **TYL** | | | |
| --- | --- | --- | --- | --- | --- | --- | --- | --- | --- | --- | --- | --- |
|  | **Mean *q*** | **Min *q*** | **Max *q*** | ***q* > 0.1** | **Mean *q*** | **Min *q*** | **Max *q*** | ***q* > 0.1** | **Mean *q*** | **Min *q*** | **Max *q*** | ***q* > 0.1** |
| AMC1B | 0.003 | 0.001 | 0.005 | 0 | 0.003 | 0.001 | 0.006 | 0 | 0.005 | 0.003 | 0.010 | 0 |
| AGS | 0.013 | 0.002 | 0.062 | 0 | 0.011 | 0.002 | 0.049 | 0 | 0.003 | 0.001 | 0.008 | 0 |
| ALB1A | 0.005 | 0.001 | 0.038 | 0 | 0.011 | 0.001 | 0.187 | 1 | 0.008 | 0.001 | 0.174 | 1 |
| AJCT3 | 0.003 | 0.001 | 0.022 | 0 | 0.002 | 0.001 | 0.004 | 0 | 0.001 | 0.001 | 0.004 | 0 |
| AGV23 | 0.018 | 0.001 | 0.279 | 3 | 0.007 | 0.002 | 0.107 | 1 | 0.003 | 0.001 | 0.012 | 0 |
| AGV21 | 0.015 | 0.002 | 0.126 | 1 | 0.004 | 0.002 | 0.011 | 0 | 0.004 | 0.002 | 0.014 | 0 |
| APHT2 | 0.006 | 0.001 | 0.032 | 0 | 0.005 | 0.001 | 0.091 | 0 | 0.003 | 0.001 | 0.024 | 0 |
| ABR | 0.007 | 0.001 | 0.103 | 1 | 0.004 | 0.001 | 0.025 | 0 | 0.002 | 0.001 | 0.012 | 0 |
| AMT4T8 | 0.005 | 0.001 | 0.123 | 1 | 0.003 | 0.001 | 0.011 | 0 | 0.004 | 0.001 | 0.023 | 0 |
| A2MT1 | 0.012 | 0.001 | 0.253 | 2 | 0.002 | 0.001 | 0.011 | 0 | 0.002 | 0.001 | 0.010 | 0 |
| ABH | 0.004 | 0.001 | 0.027 | 0 | 0.002 | 0.001 | 0.009 | 0 | 0.001 | 0.001 | 0.006 | 0 |
| ANMC | 0.005 | 0.001 | 0.083 | 0 | 0.003 | 0.001 | 0.015 | 0 | 0.003 | 0.001 | 0.011 | 0 |
| A10MC | 0.003 | 0.001 | 0.017 | 0 | 0.003 | 0.001 | 0.027 | 0 | 0.002 | 0.001 | 0.008 | 0 |
| ANR | 0.005 | 0.001 | 0.089 | 0 | 0.003 | 0.001 | 0.045 | 0 | 0.002 | 0.001 | 0.011 | 0 |
| ACH | 0.007 | 0.001 | 0.136 | 1 | 0.009 | 0.001 | 0.170 | 1 | 0.003 | 0.001 | 0.024 | 0 |
| AHH | 0.004 | 0.001 | 0.038 | 0 | 0.002 | 0.001 | 0.027 | 0 | 0.002 | 0.001 | 0.009 | 0 |
| ASH | 0.002 | 0.001 | 0.011 | 0 | 0.006 | 0.001 | 0.213 | 1 | 0.002 | 0.001 | 0.004 | 0 |
| ATH | 0.009 | 0.001 | 0.310 | 1 | 0.004 | 0.001 | 0.062 | 0 | 0.003 | 0.001 | 0.038 | 0 |
| AWC | 0.003 | 0.001 | 0.020 | 0 | 0.002 | 0.001 | 0.005 | 0 | 0.002 | 0.001 | 0.013 | 0 |
| ALRT1 | 0.014 | 0.001 | 0.549 | 1 | 0.009 | 0.001 | 0.193 | 1 | 0.004 | 0.001 | 0.036 | 0 |
| ACRT1 | 0.005 | 0.001 | 0.057 | 0 | 0.005 | 0.001 | 0.151 | 1 | 0.003 | 0.001 | 0.021 | 0 |
| ASPMC | 0.022 | 0.001 | 0.340 | 4 | 0.008 | 0.001 | 0.144 | 1 | 0.003 | 0.001 | 0.034 | 0 |
| ASPSC | 0.029 | 0.001 | 0.343 | 4 | 0.009 | 0.001 | 0.079 | 0 | 0.003 | 0.001 | 0.011 | 0 |
| ASPBC | 0.005 | 0.001 | 0.027 | 0 | 0.003 | 0.001 | 0.014 | 0 | 0.003 | 0.001 | 0.030 | 0 |
| AWR3E | 0.004 | 0.001 | 0.035 | 0 | 0.004 | 0.001 | 0.050 | 0 | 0.003 | 0.001 | 0.022 | 0 |
| ASPEC | 0.005 | 0.001 | 0.062 | 0 | 0.004 | 0.001 | 0.025 | 0 | 0.004 | 0.001 | 0.062 | 0 |
| AIBRB | 0.006 | 0.001 | 0.107 | 1 | 0.006 | 0.001 | 0.091 | 0 | 0.005 | 0.001 | 0.143 | 1 |
|  |  |  | **Total:** | **20** |  |  | **Total:** | **7** |  |  | **Total:** | **2** |
| GAB | 0.015 | 0.001 | 0.158 | 2 | 0.017 | 0.002 | 0.180 | 3 | 0.017 | 0.001 | 0.540 | 1 |
| GRCT2 | 0.002 | 0.001 | 0.009 | 0 | 0.004 | 0.001 | 0.074 | 0 | 0.003 | 0.001 | 0.020 | 0 |
| GOC | 0.003 | 0.001 | 0.033 | 0 | 0.006 | 0.001 | 0.128 | 1 | 0.004 | 0.001 | 0.028 | 0 |
| GCHT8 | 0.034 | 0.001 | 0.268 | 4 | 0.007 | 0.001 | 0.096 | 0 | 0.004 | 0.001 | 0.053 | 0 |
| GCCT8 | 0.004 | 0.001 | 0.038 | 0 | 0.008 | 0.001 | 0.290 | 1 | 0.004 | 0.001 | 0.042 | 0 |
| GWC | 0.005 | 0.001 | 0.028 | 0 | 0.006 | 0.001 | 0.068 | 0 | 0.007 | 0.001 | 0.069 | 0 |
| GDCT6 | 0.011 | 0.002 | 0.093 | 0 | 0.015 | 0.002 | 0.118 | 1 | 0.013 | 0.002 | 0.156 | 1 |
| GBQSH | 0.009 | 0.001 | 0.101 | 1 | 0.024 | 0.001 | 0.672 | 2 | 0.007 | 0.001 | 0.041 | 0 |
| GBB | 0.005 | 0.001 | 0.111 | 1 | 0.002 | 0.001 | 0.021 | 0 | 0.002 | 0.001 | 0.008 | 0 |
| GVC | 0.014 | 0.001 | 0.397 | 1 | 0.007 | 0.001 | 0.126 | 1 | 0.003 | 0.001 | 0.012 | 0 |
| GCCEC | 0.022 | 0.001 | 0.285 | 3 | 0.064 | 0.001 | 0.874 | 5 | 0.004 | 0.001 | 0.023 | 0 |
| GSBT4 | 0.002 | 0.001 | 0.008 | 0 | 0.003 | 0.001 | 0.020 | 0 | 0.003 | 0.001 | 0.010 | 0 |
| GWC11 | 0.004 | 0.001 | 0.026 | 0 | 0.005 | 0.001 | 0.072 | 0 | 0.004 | 0.001 | 0.044 | 0 |
| GTB | 0.007 | 0.001 | 0.070 | 0 | 0.005 | 0.001 | 0.054 | 0 | 0.004 | 0.001 | 0.019 | 0 |
| GSB | 0.001 | 0.001 | 0.003 | 0 | 0.002 | 0.001 | 0.008 | 0 | 0.002 | 0.001 | 0.013 | 0 |
| GHG | 0.006 | 0.001 | 0.088 | 0 | 0.005 | 0.001 | 0.039 | 0 | 0.005 | 0.001 | 0.066 | 0 |
| GC28B | 0.002 | 0.001 | 0.011 | 0 | 0.003 | 0.001 | 0.016 | 0 | 0.003 | 0.001 | 0.015 | 0 |
| GSB1 | 0.003 | 0.001 | 0.013 | 0 | 0.004 | 0.001 | 0.042 | 0 | 0.008 | 0.001 | 0.161 | 1 |
| GMC1 | 0.003 | 0.001 | 0.018 | 0 | 0.004 | 0.001 | 0.036 | 0 | 0.005 | 0.001 | 0.054 | 0 |
| GMC | 0.004 | 0.001 | 0.064 | 0 | 0.004 | 0.001 | 0.057 | 0 | 0.003 | 0.001 | 0.018 | 0 |
| G2213 | 0.004 | 0.001 | 0.013 | 0 | 0.004 | 0.001 | 0.056 | 0 | 0.003 | 0.001 | 0.030 | 0 |
| GPG | 0.004 | 0.001 | 0.024 | 0 | 0.002 | 0.001 | 0.008 | 0 | 0.002 | 0.001 | 0.015 | 0 |
| GRGC | 0.003 | 0.001 | 0.014 | 0 | 0.002 | 0.001 | 0.006 | 0 | 0.003 | 0.001 | 0.012 | 0 |
| GHI | 0.019 | 0.001 | 0.292 | 2 | 0.025 | 0.001 | 0.633 | 1 | 0.004 | 0.001 | 0.017 | 0 |
|  |  |  | **Total:** | **14** |  |  | **Total:** | **15** |  |  | **Total:** | **3** |
|  |  |  |  |  |  |  |  |  |  |  |  |  |
| GCC | 0.002 | 0.001 | 0.018 | 0 | 0.002 | 0.001 | 0.010 | 0 | 0.004 | 0.001 | 0.030 | 0 |
|  |  |  |  |  |  |  |  |  |  |  |  |  |
| GU112 | 0.001 | 0.001 | 0.003 | 0 | 0.002 | 0.001 | 0.003 | 0 | 0.002 | 0.001 | 0.005 | 0 |
|  |  |  |  |  |  |  |  |  |  |  |  |  |
|  |  |  |  |  |  |  |  |  |  |  |  |  |
| SCCT5 | 0.003 | 0.001 | 0.048 | 0 |  |  |  |  |  |  |  |  |
| SCT3B | 0.001 | 0.001 | 0.005 | 0 |  |  |  |  |  |  |  |  |
| SUT58 | 0.003 | 0.001 | 0.015 | 0 |  |  |  |  |  |  |  |  |
| SUT56 | 0.004 | 0.001 | 0.032 | 0 |  |  |  |  |  |  |  |  |
| SCR | 0.006 | 0.001 | 0.088 | 0 |  |  |  |  |  |  |  |  |
| SEWC | 0.017 | 0.001 | 0.440 | 2 |  |  |  |  |  |  |  |  |
| SU482 | 0.007 | 0.001 | 0.140 | 1 |  |  |  |  |  |  |  |  |
| SKC | 0.005 | 0.001 | 0.043 | 0 |  |  |  |  |  |  |  |  |
| SLC | 0.004 | 0.001 | 0.029 | 0 |  |  |  |  |  |  |  |  |
| SAHB | 0.003 | 0.001 | 0.014 | 0 |  |  |  |  |  |  |  |  |
| SWC | 0.002 | 0.001 | 0.010 | 0 |  |  |  |  |  |  |  |  |
| SCC | 0.002 | 0.001 | 0.020 | 0 |  |  |  |  |  |  |  |  |
| SU3811 | 0.002 | 0.001 | 0.032 | 0 |  |  |  |  |  |  |  |  |
| SCB | 0.004 | 0.001 | 0.070 | 0 |  |  |  |  |  |  |  |  |
| SDR | 0.002 | 0.001 | 0.010 | 0 |  |  |  |  |  |  |  |  |
| SBC | 0.002 | 0.001 | 0.006 | 0 |  |  |  |  |  |  |  |  |
|  |  |  | **Total:** | **3** |  |  |  |  |  |  |  |  |
|  |  |  |  |  |  |  |  |  |  |  |  |  |
| ESBT3 | 0.010 | 0.001 | 0.101 | 1 |  |  |  |  |  |  |  |  |
| EC34A | 0.010 | 0.001 | 0.261 | 1 |  |  |  |  |  |  |  |  |
| EGCT3 | 0.012 | 0.001 | 0.363 | 1 |  |  |  |  |  |  |  |  |
| ESCT5 | 0.021 | 0.001 | 0.464 | 3 |  |  |  |  |  |  |  |  |
| EEBCT | 0.012 | 0.001 | 0.242 | 1 |  |  |  |  |  |  |  |  |
| ECT6A | 0.002 | 0.001 | 0.004 | 0 |  |  |  |  |  |  |  |  |
|  |  |  | **Total:** | **7** |  |  |  |  |  |  |  |  |

**Table A4** Membership proportions (*q*-values) of wild Brook Trout to hatchery strains, produced with the population-specific STRUCTURE analysis using the default parameter set. The mean, minimum, and maximum *q*-values to each hatchery cluster are reported for each, along with the number of individuals displaying *q*-values > 0.1, indicating potential hatchery introgression.

| **ID** | **ROM** | | | | **OSW** | | | | **TYL** | | | |
| --- | --- | --- | --- | --- | --- | --- | --- | --- | --- | --- | --- | --- |
|  | **Mean *q*** | **Min *q*** | **Max *q*** | ***q* > 0.1** | **Mean *q*** | **Min *q*** | **Max *q*** | ***q* > 0.1** | **Mean *q*** | **Min *q*** | **Max *q*** | ***q* > 0.1** |
| AMC1B | 0.006 | 0.002 | 0.013 | 0 | 0.005 | 0.002 | 0.014 | 0 | 0.005 | 0.003 | 0.007 | 0 |
| AGS | 0.016 | 0.003 | 0.077 | 0 | 0.015 | 0.003 | 0.064 | 0 | 0.004 | 0.002 | 0.012 | 0 |
| ALB1A | 0.008 | 0.002 | 0.064 | 0 | 0.013 | 0.002 | 0.174 | 1 | 0.010 | 0.002 | 0.203 | 1 |
| AJCT3 | 0.005 | 0.002 | 0.034 | 0 | 0.003 | 0.002 | 0.020 | 0 | 0.002 | 0.001 | 0.008 | 0 |
| AGV23 | 0.023 | 0.002 | 0.301 | 3 | 0.010 | 0.002 | 0.117 | 1 | 0.004 | 0.002 | 0.018 | 0 |
| AGV21 | 0.024 | 0.002 | 0.197 | 1 | 0.007 | 0.002 | 0.024 | 0 | 0.005 | 0.002 | 0.014 | 0 |
| APHT2 | 0.010 | 0.002 | 0.041 | 0 | 0.020 | 0.002 | 0.342 | 2 | 0.006 | 0.001 | 0.057 | 0 |
| ABR | 0.010 | 0.002 | 0.131 | 1 | 0.006 | 0.002 | 0.035 | 0 | 0.003 | 0.002 | 0.015 | 0 |
| AMT4T8 | 0.008 | 0.001 | 0.158 | 1 | 0.006 | 0.001 | 0.044 | 0 | 0.006 | 0.001 | 0.047 | 0 |
| A2MT1 | 0.014 | 0.001 | 0.279 | 2 | 0.004 | 0.001 | 0.042 | 0 | 0.004 | 0.001 | 0.057 | 0 |
| ABH | 0.005 | 0.002 | 0.038 | 0 | 0.003 | 0.002 | 0.013 | 0 | 0.002 | 0.001 | 0.009 | 0 |
| ANMC | 0.008 | 0.002 | 0.110 | 1 | 0.006 | 0.001 | 0.056 | 0 | 0.005 | 0.001 | 0.021 | 0 |
| A10MC | 0.005 | 0.001 | 0.034 | 0 | 0.006 | 0.001 | 0.057 | 0 | 0.003 | 0.001 | 0.012 | 0 |
| ANR | 0.012 | 0.002 | 0.176 | 2 | 0.007 | 0.001 | 0.089 | 0 | 0.005 | 0.001 | 0.087 | 0 |
| ACH | 0.009 | 0.001 | 0.121 | 1 | 0.012 | 0.001 | 0.153 | 2 | 0.005 | 0.001 | 0.039 | 0 |
| AHH | 0.009 | 0.001 | 0.073 | 0 | 0.010 | 0.001 | 0.189 | 1 | 0.003 | 0.001 | 0.019 | 0 |
| ASH | 0.004 | 0.001 | 0.024 | 0 | 0.012 | 0.001 | 0.399 | 1 | 0.003 | 0.001 | 0.012 | 0 |
| ATH | 0.010 | 0.002 | 0.209 | 1 | 0.009 | 0.001 | 0.134 | 1 | 0.004 | 0.001 | 0.036 | 0 |
| AWC | 0.004 | 0.001 | 0.030 | 0 | 0.003 | 0.002 | 0.015 | 0 | 0.004 | 0.001 | 0.043 | 0 |
| ALRT1 | 0.016 | 0.001 | 0.523 | 1 | 0.013 | 0.001 | 0.261 | 1 | 0.006 | 0.001 | 0.050 | 0 |
| ACRT1 | 0.008 | 0.001 | 0.120 | 1 | 0.008 | 0.001 | 0.214 | 1 | 0.005 | 0.001 | 0.062 | 0 |
| ASPMC | 0.031 | 0.001 | 0.364 | 4 | 0.012 | 0.001 | 0.191 | 1 | 0.005 | 0.001 | 0.049 | 0 |
| ASPSC | 0.031 | 0.002 | 0.342 | 5 | 0.014 | 0.001 | 0.126 | 3 | 0.004 | 0.001 | 0.016 | 0 |
| ASPBC | 0.007 | 0.001 | 0.044 | 0 | 0.004 | 0.001 | 0.019 | 0 | 0.004 | 0.001 | 0.042 | 0 |
| AWR3E | 0.007 | 0.001 | 0.056 | 0 | 0.008 | 0.001 | 0.131 | 1 | 0.005 | 0.001 | 0.048 | 0 |
| ASPEC | 0.008 | 0.002 | 0.089 | 0 | 0.006 | 0.001 | 0.047 | 0 | 0.006 | 0.001 | 0.102 | 1 |
| AIBRB | 0.011 | 0.001 | 0.172 | 1 | 0.011 | 0.002 | 0.143 | 2 | 0.008 | 0.001 | 0.163 | 1 |
|  |  |  | **Total:** | **25** |  |  | **Total:** | **18** |  |  | **Total:** | **3** |
| GAB | 0.016 | 0.002 | 0.149 | 2 | 0.024 | 0.002 | 0.268 | 4 | 0.015 | 0.002 | 0.349 | 1 |
| GRCT2 | 0.004 | 0.001 | 0.015 | 0 | 0.007 | 0.001 | 0.176 | 1 | 0.005 | 0.001 | 0.034 | 0 |
| GOC | 0.004 | 0.001 | 0.034 | 0 | 0.008 | 0.001 | 0.135 | 1 | 0.005 | 0.001 | 0.037 | 0 |
| GCHT8 | 0.045 | 0.001 | 0.377 | 5 | 0.011 | 0.002 | 0.161 | 1 | 0.005 | 0.001 | 0.043 | 0 |
| GCCT8 | 0.006 | 0.001 | 0.071 | 0 | 0.012 | 0.001 | 0.367 | 1 | 0.005 | 0.001 | 0.045 | 0 |
| GWC | 0.006 | 0.001 | 0.030 | 0 | 0.007 | 0.002 | 0.061 | 0 | 0.009 | 0.002 | 0.097 | 0 |
| GDCT6 | 0.011 | 0.002 | 0.092 | 0 | 0.015 | 0.002 | 0.118 | 1 | 0.013 | 0.002 | 0.155 | 1 |
| GBQSH | 0.013 | 0.002 | 0.133 | 2 | 0.026 | 0.002 | 0.544 | 2 | 0.009 | 0.002 | 0.059 | 0 |
| GBB | 0.007 | 0.001 | 0.154 | 1 | 0.006 | 0.001 | 0.115 | 1 | 0.003 | 0.001 | 0.015 | 0 |
| GVC | 0.018 | 0.002 | 0.413 | 1 | 0.010 | 0.002 | 0.159 | 1 | 0.005 | 0.002 | 0.018 | 0 |
| GCCEC | 0.023 | 0.002 | 0.256 | 3 | 0.065 | 0.002 | 0.699 | 7 | 0.006 | 0.002 | 0.044 | 0 |
| GSBT4 | 0.004 | 0.002 | 0.013 | 0 | 0.005 | 0.002 | 0.058 | 0 | 0.004 | 0.002 | 0.026 | 0 |
| GWC11 | 0.008 | 0.002 | 0.073 | 0 | 0.010 | 0.002 | 0.187 | 1 | 0.005 | 0.001 | 0.046 | 0 |
| GTB | 0.009 | 0.002 | 0.080 | 0 | 0.008 | 0.002 | 0.076 | 0 | 0.005 | 0.001 | 0.025 | 0 |
| GSB | 0.002 | 0.001 | 0.008 | 0 | 0.003 | 0.001 | 0.026 | 0 | 0.004 | 0.001 | 0.044 | 0 |
| GHG | 0.008 | 0.001 | 0.116 | 1 | 0.007 | 0.002 | 0.048 | 0 | 0.007 | 0.002 | 0.078 | 0 |
| GC28B | 0.004 | 0.002 | 0.014 | 0 | 0.006 | 0.002 | 0.035 | 0 | 0.004 | 0.002 | 0.028 | 0 |
| GSB1 | 0.005 | 0.002 | 0.026 | 0 | 0.007 | 0.002 | 0.058 | 0 | 0.011 | 0.002 | 0.194 | 1 |
| GMC1 | 0.005 | 0.001 | 0.028 | 0 | 0.008 | 0.002 | 0.050 | 0 | 0.008 | 0.002 | 0.067 | 0 |
| GMC | 0.007 | 0.001 | 0.124 | 1 | 0.008 | 0.002 | 0.107 | 1 | 0.004 | 0.002 | 0.021 | 0 |
| G2213 | 0.006 | 0.002 | 0.018 | 0 | 0.007 | 0.002 | 0.097 | 0 | 0.005 | 0.002 | 0.026 | 0 |
| GPG | 0.008 | 0.002 | 0.078 | 0 | 0.004 | 0.002 | 0.029 | 0 | 0.004 | 0.002 | 0.045 | 0 |
| GRGC | 0.007 | 0.002 | 0.064 | 0 | 0.003 | 0.002 | 0.009 | 0 | 0.004 | 0.002 | 0.018 | 0 |
| GHI | 0.018 | 0.002 | 0.200 | 2 | 0.032 | 0.002 | 0.717 | 1 | 0.006 | 0.002 | 0.025 | 0 |
|  |  |  | **Total:** | **18** |  |  | **Total:** | **23** |  |  | **Total:** | **3** |
|  |  |  |  |  |  |  |  |  |  |  |  |  |
| GCC | 0.004 | 0.001 | 0.026 | 0 | 0.004 | 0.002 | 0.023 | 0 | 0.006 | 0.002 | 0.074 | 0 |
|  |  |  |  |  |  |  |  |  |  |  |  |  |
| GU112 | 0.002 | 0.001 | 0.011 | 0 | 0.003 | 0.001 | 0.007 | 0 | 0.003 | 0.002 | 0.006 | 0 |
|  |  |  |  |  |  |  |  |  |  |  |  |  |
|  |  |  |  |  |  |  |  |  |  |  |  |  |
| SCCT5 | 0.005 | 0.002 | 0.095 | 0 |  |  |  |  |  |  |  |  |
| SCT3B | 0.004 | 0.001 | 0.024 | 0 |  |  |  |  |  |  |  |  |
| SUT58 | 0.006 | 0.001 | 0.045 | 0 |  |  |  |  |  |  |  |  |
| SUT56 | 0.009 | 0.001 | 0.075 | 0 |  |  |  |  |  |  |  |  |
| SCR | 0.009 | 0.002 | 0.124 | 1 |  |  |  |  |  |  |  |  |
| SEWC | 0.018 | 0.002 | 0.309 | 2 |  |  |  |  |  |  |  |  |
| SU482 | 0.011 | 0.002 | 0.174 | 1 |  |  |  |  |  |  |  |  |
| SKC | 0.008 | 0.002 | 0.051 | 0 |  |  |  |  |  |  |  |  |
| SLC | 0.008 | 0.002 | 0.048 | 0 |  |  |  |  |  |  |  |  |
| SAHB | 0.006 | 0.002 | 0.039 | 0 |  |  |  |  |  |  |  |  |
| SWC | 0.005 | 0.002 | 0.030 | 0 |  |  |  |  |  |  |  |  |
| SCC | 0.006 | 0.001 | 0.046 | 0 |  |  |  |  |  |  |  |  |
| SU3811 | 0.004 | 0.001 | 0.036 | 0 |  |  |  |  |  |  |  |  |
| SCB | 0.010 | 0.002 | 0.131 | 1 |  |  |  |  |  |  |  |  |
| SDR | 0.005 | 0.001 | 0.023 | 0 |  |  |  |  |  |  |  |  |
| SBC | 0.004 | 0.001 | 0.019 | 0 |  |  |  |  |  |  |  |  |
|  |  |  | **Total:** | **5** |  |  |  |  |  |  |  |  |
|  |  |  |  |  |  |  |  |  |  |  |  |  |
| ESBT3 | 0.016 | 0.002 | 0.123 | 2 |  |  |  |  |  |  |  |  |
| EC34A | 0.018 | 0.003 | 0.333 | 1 |  |  |  |  |  |  |  |  |
| EGCT3 | 0.018 | 0.002 | 0.376 | 1 |  |  |  |  |  |  |  |  |
| ESCT5 | 0.018 | 0.002 | 0.378 | 2 |  |  |  |  |  |  |  |  |
| EEBCT | 0.025 | 0.002 | 0.304 | 5 |  |  |  |  |  |  |  |  |
| ECT6A | 0.003 | 0.002 | 0.008 | 0 |  |  |  |  |  |  |  |  |
|  |  |  | **Total:** | **11** |  |  |  |  |  |  |  |  |
