# Supplementary material for: Genetic diversity, admixture, and hatchery influence in Brook Trout (Salvelinus fontinalis) throughout western New York State
Source: Ecol Evol. 2019 Jun 14;9(13):7455–79. doi: 10.1002/ece3.5237 (PMC6635958; doi:10.1002/ece3.5237)
Supplement: Supplementary file 2 [file ECE3-9-7455-s002.pdf]

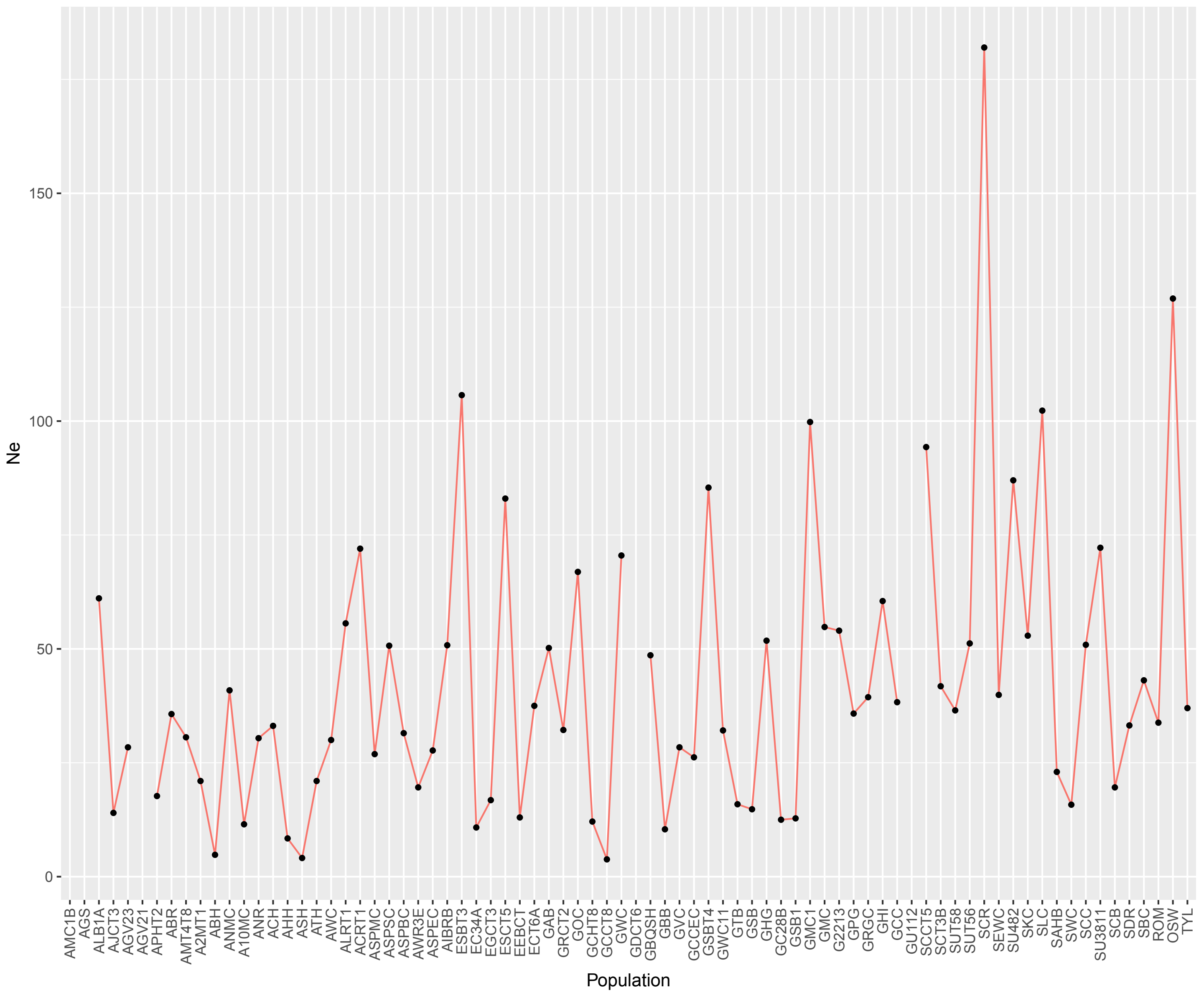

Figure A1 Line graph showing the mean effective population size estimates (N_e) for wild Brook Trout populations in western New York State and the mean effective number of breeders (N_b) for hatchery Brook Trout strains. See Table 2 for confidence intervals.

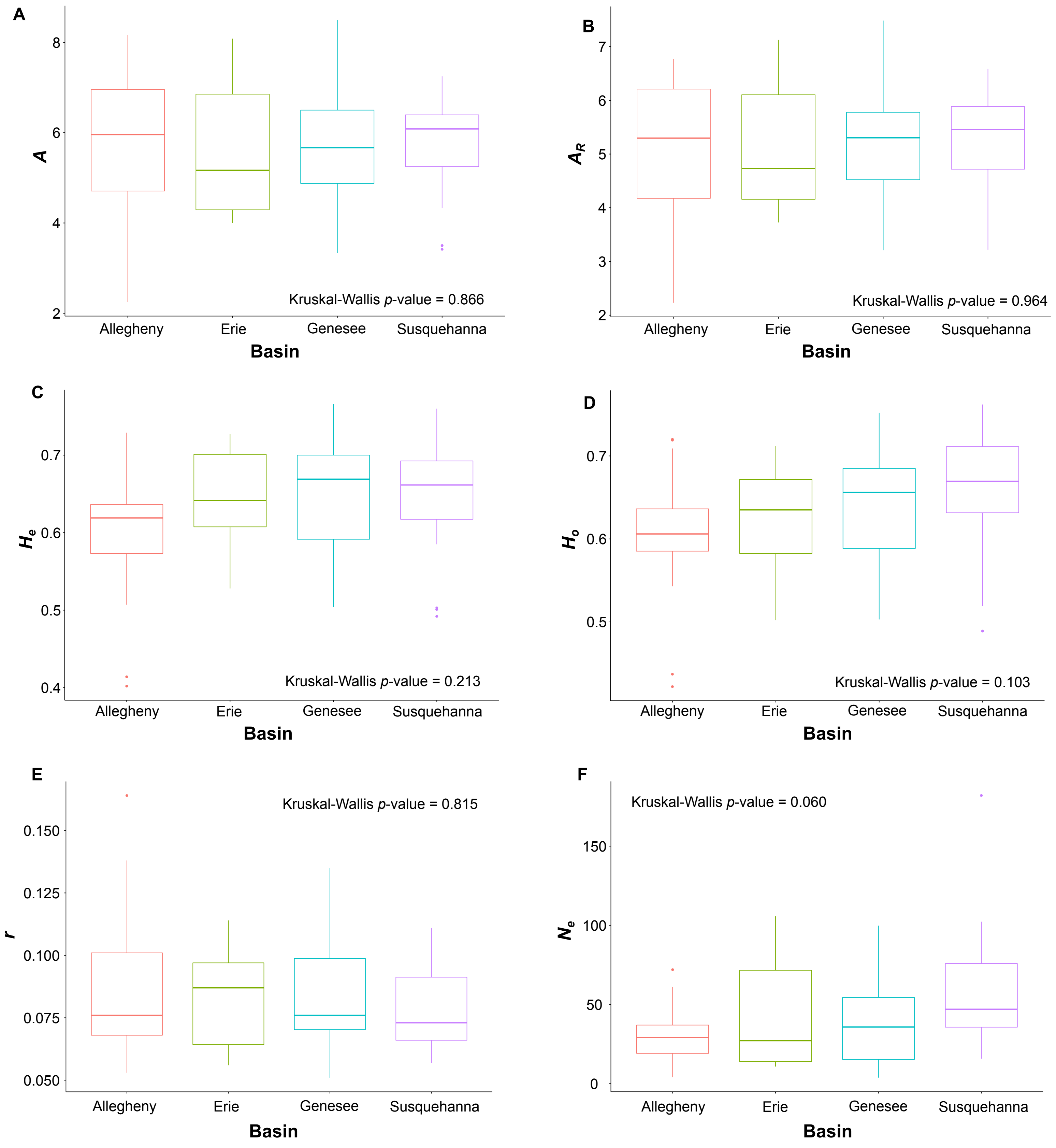

Figure A2 Box plots of diversity metrics for each major basin; (A) average number of alleles per locus, A; (B) allelic richness, A_R; (C) expected heterozygosity, H_e; (D) observed heterozygosity, H_o; (E) maximum-likelihood estimates of relatedness, r; (F) effective population size, N_e.

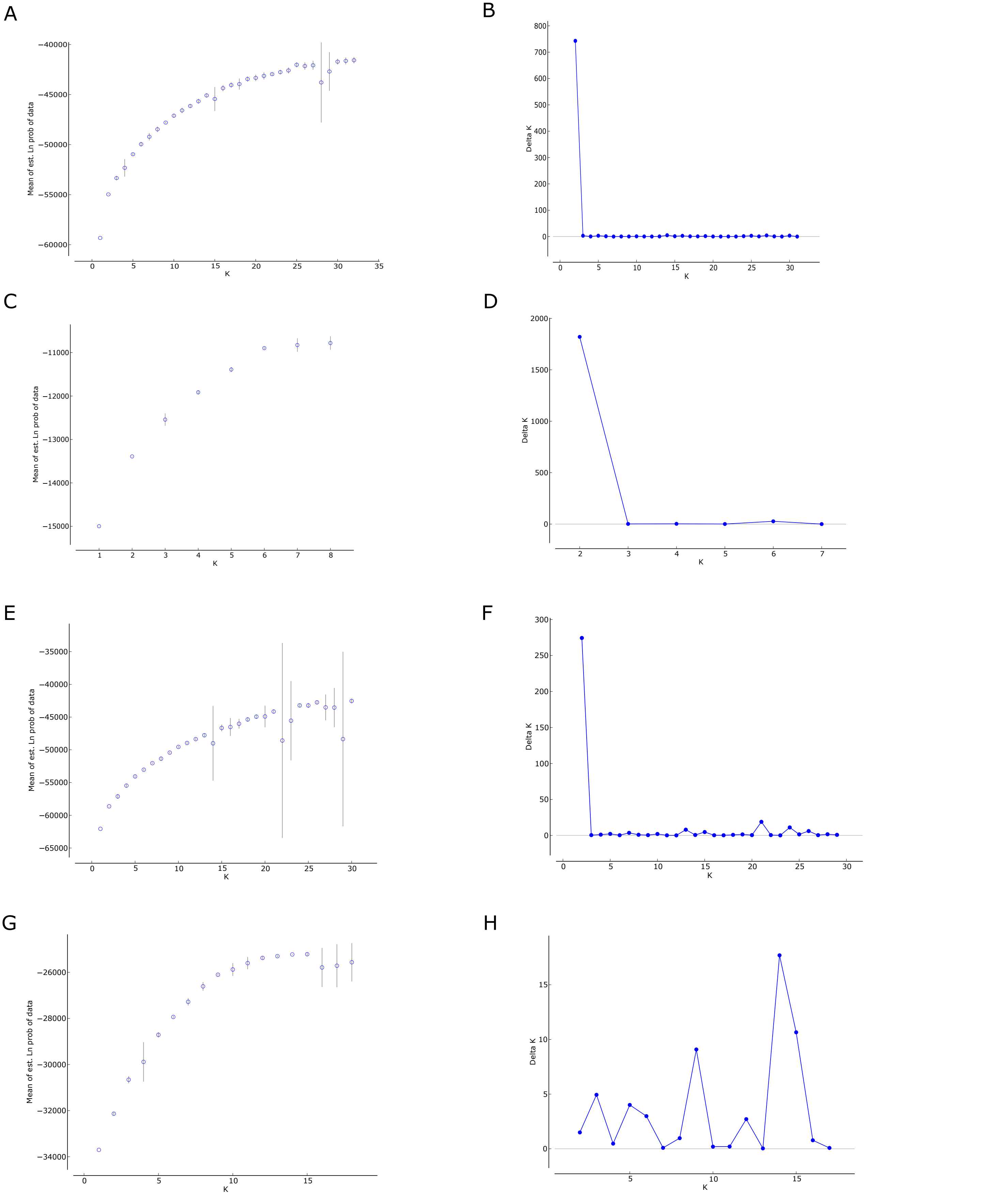

Figure A3 Ad-hoc analysis of the STRUCTURE results, using the default parameters, to identify the optimal *K* value for each data set. For each drainage basin, the Pritchard et al. (2000) method is shown on the left (A, C, E, G), and the Evanno et al. (2005) method is shown on the right (B, D, F, H). **(A/B)**: the Allegheny basin data set; **(C/D)**: the Erie/Niagara basin data set; **(E/F)**: the Genesee basin data set; and **(G/H)**: the Susquehanna basin data set.

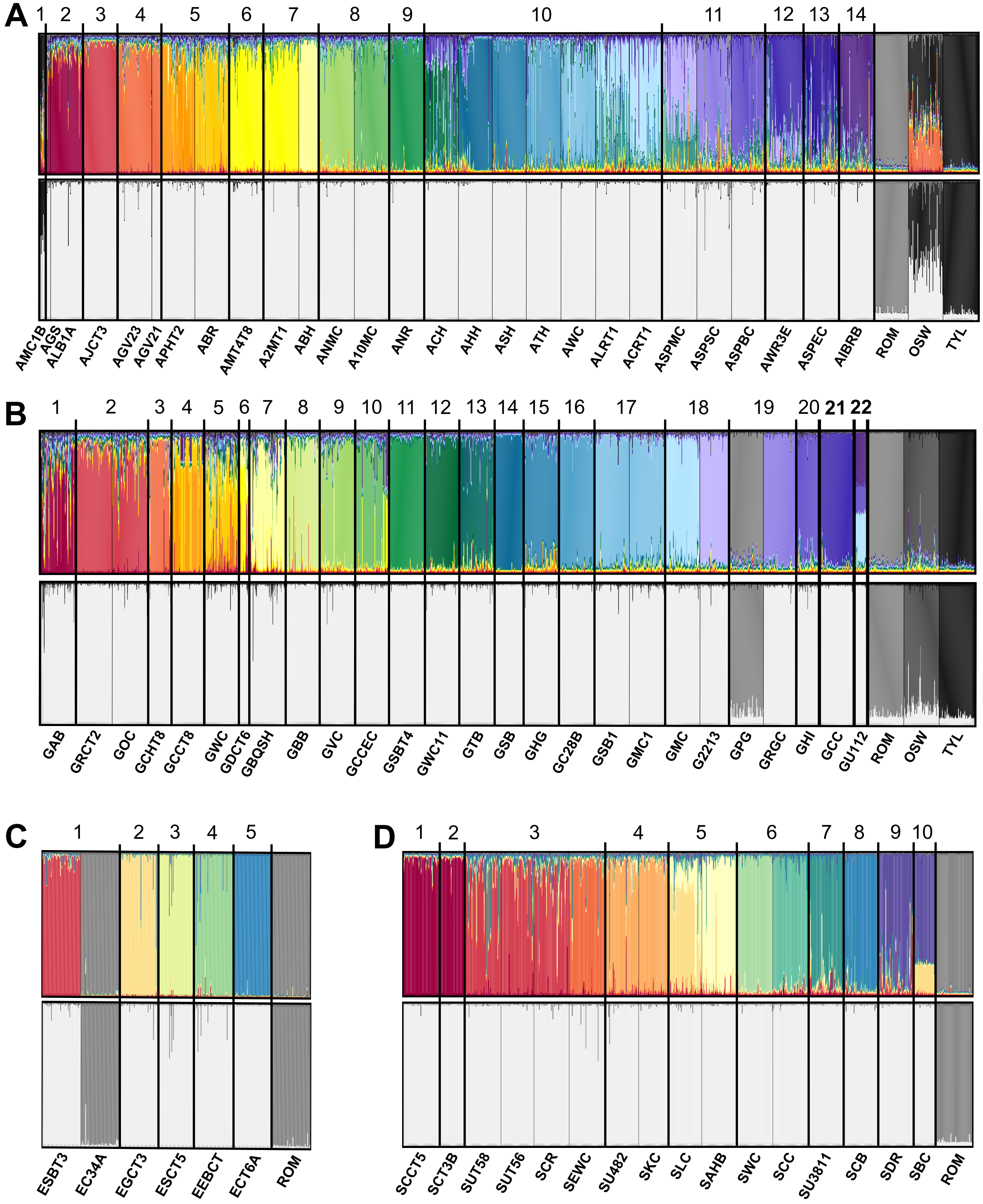

Figure A4 Bayesian clustering analysis of Brook Trout performed using the default parameter set, showing separate sampling localities labeled with their associated population ID, and subwatershed (12-digit HUC level) delineations numbered corresponding to Table 1. For each basin, the full color plot is shown (top) as well as a plot with the wild Brook Trout genetic clusters all depicted in light gray (bottom) to better visualize hatchery contibutions. **(A)** Allegheny: $K = 25$, **(B)** Genesee: $K = 26$, **(C)** Erie/Niagara: $K = 6$, and **(D)** Susquehanna: $K = 12$.

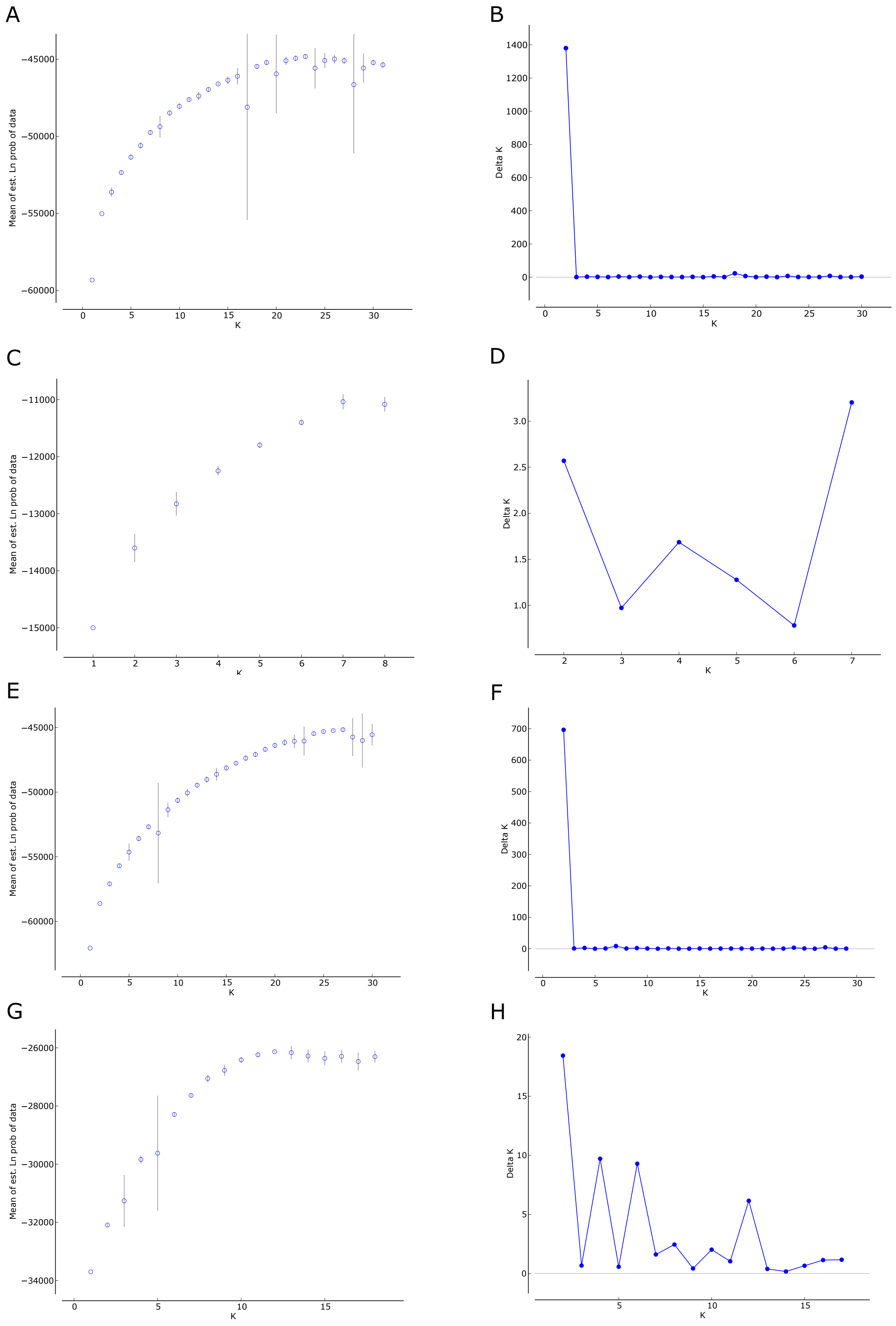

Figure A5 Ad-hoc analysis of the STRUCTURE results, using the alternative parameters, to identify the optimal *K* value for each data set. For each drainage basin, the Pritchard et al. (2000) method is shown on the left (A, C, E, G), and the Evanno et al. (2005) method is shown on the right (B, D, F, H). **(A/B)**: the Allegheny basin data set; **(C/D)**: the Erie/Niagara basin data set; **(E/F)**: the Genesee basin data set; and **(G/H)**: the Susquehanna basin data set.

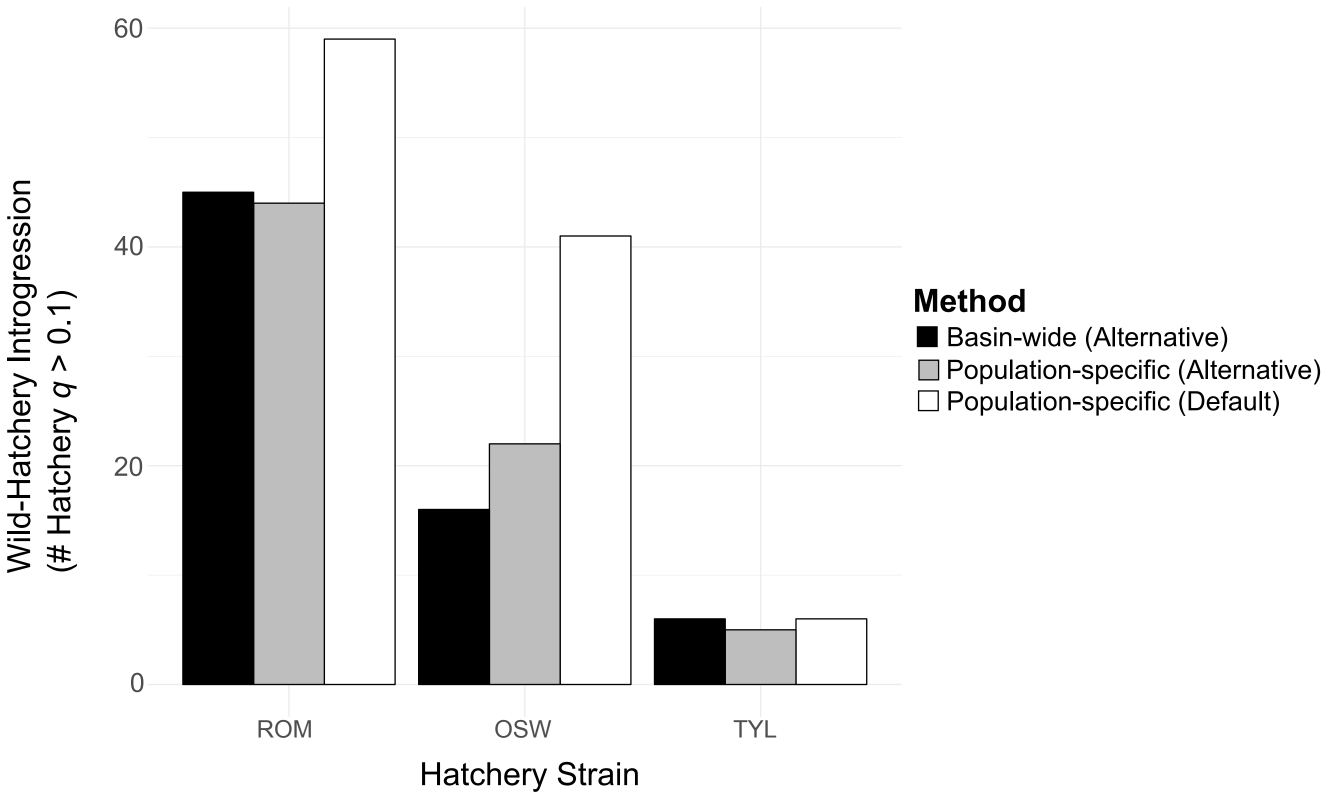

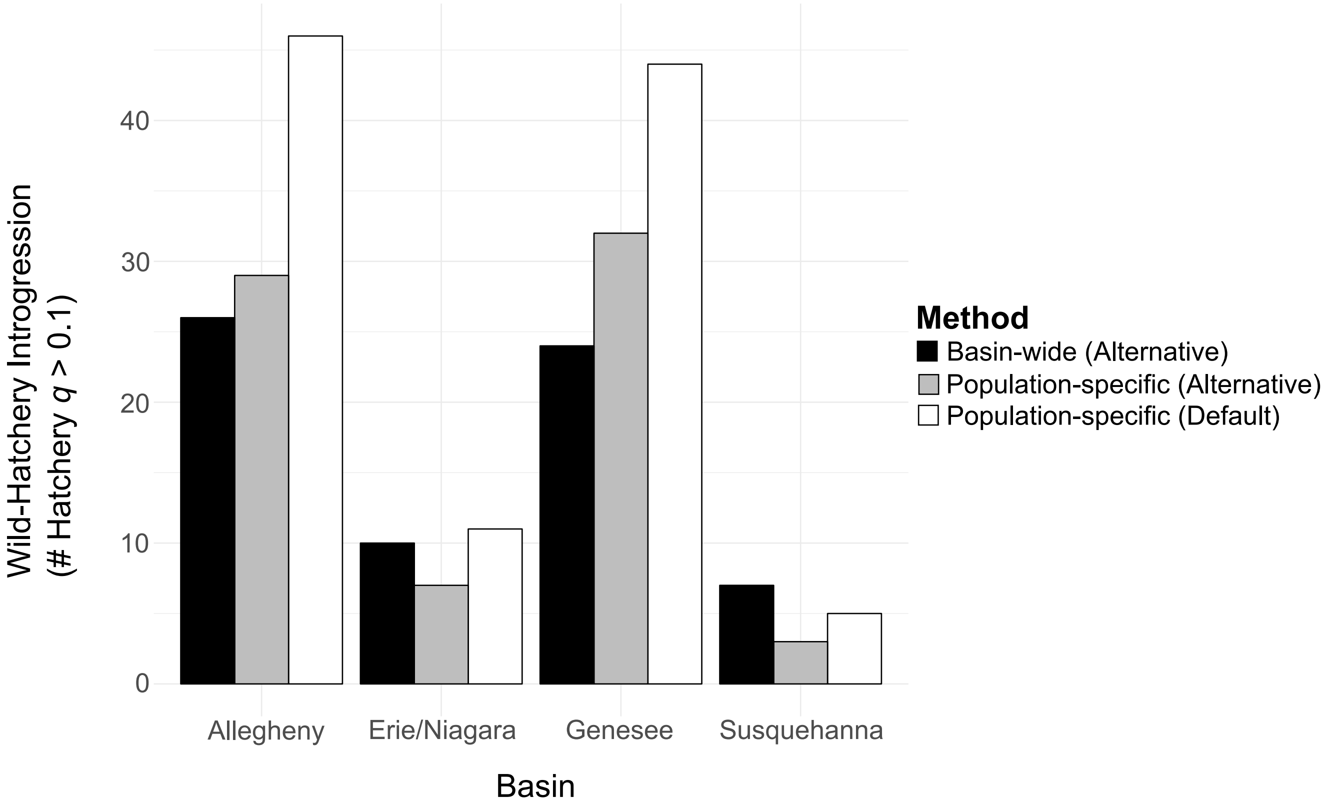

Figure A6 Bar graphs comparing the three methods of estimating wild-hatchery introgression, the basin-wide STRUCTURE analysis using the alternative parameter set, the population-specific STRUCTURE analysis using the alternative parameter set, and the population-specific STRUCTURE analysis using the default parameter set. The y-axis represents the total number of individuals showing wild-hatchery introgression (*q*-values to a hatchery cluster > 0.1) for each hatchery strain (**top**) and for each major drainage basin (**bottom**).

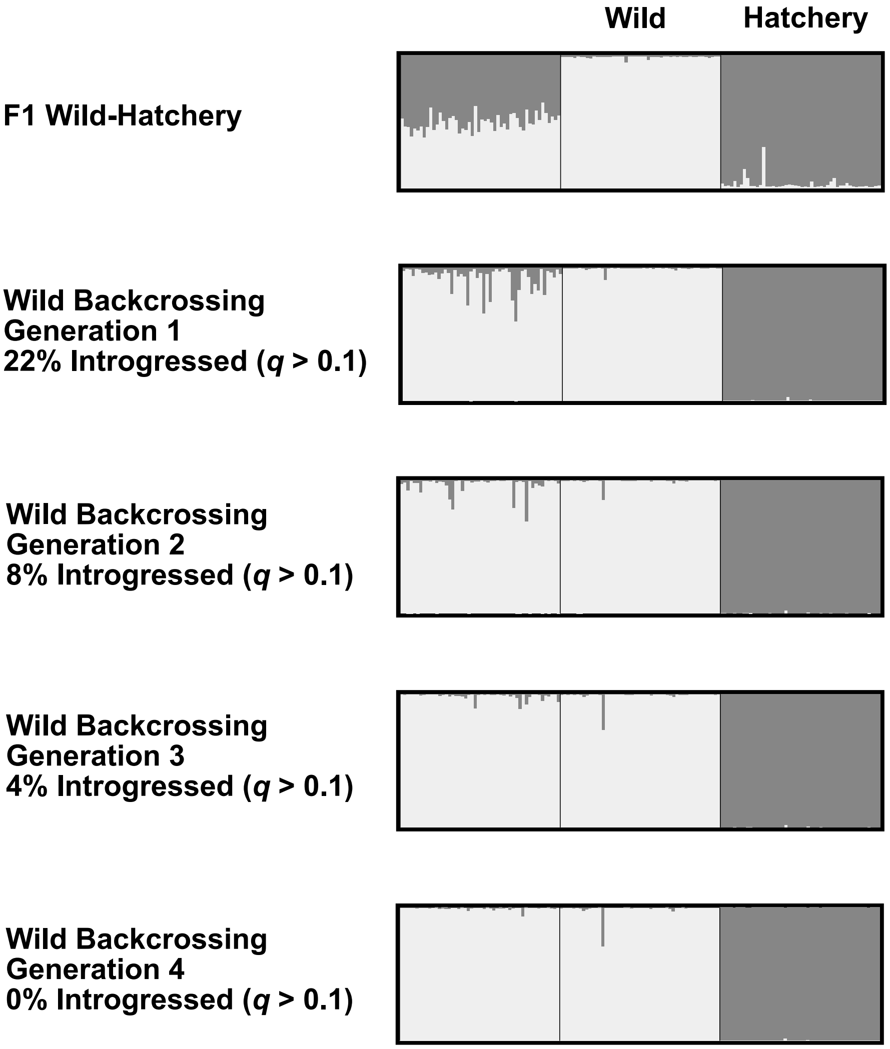

Figure A7 STRUCTURE plots showing the results of the simulated wild-hatchery introgression and backcrossing analysis. The y-axis represents the membership proportions (q-values) to each of the genetic clusters – wild shown in light gray and hatchery shown in dark gray.
